# Continuous Flow Paper Spray Ionization Mass Spectrometry for In-Depth Characterization of Anticancer Drugs in Tissues: Addressing Mass Spectral Complexity

**DOI:** 10.1021/jasms.5c00374

**Published:** 2026-01-16

**Authors:** Pallab Basuri, Konrad Klinghammer, Oliver Klein, Dietrich A. Volmer

**Affiliations:** † Department of Bioanalytical Chemistry, Institute of Chemistry, 9373 Humboldt-Universität zu Berlin, 12489 Berlin, Germany; ‡ Hematology, Oncology and Tumor Immunology, Charité Universitätsmedizin, 12200 Berlin, Germany; § Center for Regenerative Therapies, Core Unit Imaging Mass Spectrometry, Charité Universitätsmedizin, 13353 Berlin, Germany

**Keywords:** continuous, flow, paper, mass, CFPSI, rapid, anticancer, drugs, tissue, samples., analysis, spectral

## Abstract

We introduced continuous flow paper spray ionization mass spectrometry (CFPSI MS) for the rapid detection and characterization of anticancer drugs in solid tissue samples. CFPSI is a paper spray-based semiquantitative method using continuous flow of an internal standard to quantify the amounts of drugs released from the tissue samples. Using patient-derived xenograft (PDX) mouse model tissue samples, we observed differential absorption of three anticancer drugs, palbociclib, copanlisib, and olaparib. Palbociclib was found to be bioabsorbed in the tissue samples to the largest extent. Tandem mass spectrometric analysis explored the in-source chemical reactivity of these drugs, leading to significant spectral complexity. Our findings highlight the importance of careful spectral interpretation in complex biological matrices and support the development of future rapid quantitative CFPSI analysis of these drugs in tissue samples.

## Graphical abstract

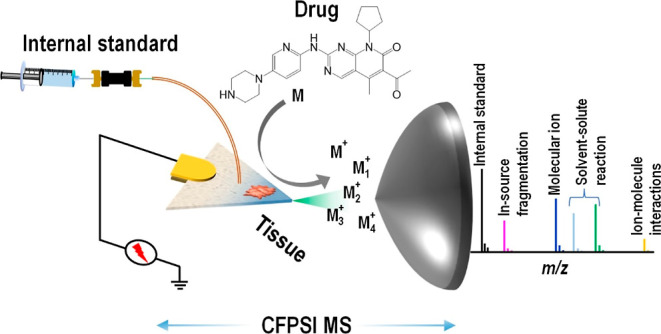

## Introduction

Understanding the absorption efficiency of drugs in tissues could aid the development of new drug molecules, help improve drug delivery methods, and enable more precise pharmacokinetic projections.^1,2^ This is crucial to developing and guiding personalized treatment methods. However, rapid and quantitative analysis of these drugs in biological tissue and biofluids such as blood, and urine is a key analytical challenge.^3,4^ Limited availability of appropriate tissue samples often limits the analysis of drug absorption kinetics and its distribution. To overcome this limitation, model tissues such as patient-derived xenograft (PDX) models, in which tumor tissues from patients are implanted into immunocompromised or humanized mice, have been introduced.^5,6^ These tissue models have shown superiority in reproducing the characteristics of cancer, such as the spatial structure and the intratumor heterogeneity.^5,7^ Moreover, PDX models retain the genomic features of patients across different stages, subtypes, and diversified treatment backgrounds.^8,9^ Analyzing drug content in PDX model tissue samples can lead to the understanding of the absorption efficiency of drugs.^10^

Mass spectrometry (MS) has become the gold standard technique for the sensitive detection and quantification of molecules in biological samples,^11^ in particular electrospray ionization (ESI) MS in combination with liquid chromatography.^12^ However, major challenges remain, such as the complexity of sample preparation,^13^ efficient analyte extraction,^14^ preconcentration,^4^ derivatization,^15^ and instrumental method development.^16^ By enabling molecular ionization directly from a substance, with little to no modification, ambient ionization techniques such as desorption electrospray ionization (DESI),^17^ paper spray ionization (PSI),^18^ low temperature plasma (LTP),^19^ liquid extraction surface analysis (LESA), and surface-assisted laser desorption ionization (SALDI)^20^ have helped in overcoming some of these limitations.

Among these ambient ionization methods, PSI MS provides a low cost and rapid detection variant.^21^ It generally involves a triangular paper connected to a high-voltage DC power supply to trigger ionization of molecules in the form of charged microdroplets. These droplets release bare ions in the gas phase during their flight toward the inlet of the MS. Different variants of PSI MS exist including ionization from cotton, leaf, needle, blade, etc.^22–24^ Some variants also use light,^25^ sound,^26^ and chemical energy^27^ in combination with or without the application of electrical energy. Quantitative paper spray methods have also been demonstrated in the literature; however, they are often limited by signal instabilities and reproducibility.^28^ The choice of proper solvents, additives, and source parameters is crucial for any spray-based MS experiments. In-source fragmentation (ISF) and chemical transformation of functional groups, ion clustering reactions, and solvent–solute interactions can lead to increased spectral complexity and inaccurate quantification during analysis. A recent study indicates that ISF alone may explain over 70% of the peaks present in typical liquid chromatography–mass spectrometry (LC–MS)/MS experiments, highlighting its significance.^29^ Other recent reports also indicate that electrospray droplets can facilitate chemical reactions that would otherwise be impossible in a condensed phase^30^ due to their unique physicochemical properties such as interfacial pH,^31^ molecular enrichments,^32^ high surface charge density,^33^ and limited solvation.^34^ In principle, multifunctional molecules may undergo chemical derivatization with neighboring molecules and solvents used for ionization. Furthermore, these droplets can even enable intramolecular rearrangements, expanding the potential for complex reactions and transformations, leading to complexity in the mass spectra. On the other hand, for quantitative analysis, often, internal standards are codeposited with the sample on the paper surface, which can cause nonuniform desorption or elution of molecules, influencing relative intensities of the peaks.^35^ A few modifications of PSI have been demonstrated, involving a continuous supply of solvents, to enhance signal stability and duration.^35,36^ However, PSI MS still requires improvements for its effective implementation in clinical practice.

Recently, using ion mobility MS, we found that anticancer drugs such as palbociclib, copanlisib, and olaparib (Figure 1a–c) exhibit formation of protomers during ESI, which may complicate quantitative analysis using LC–MS/MS.^37^ In this study, we demonstrate the potential of continuous flow paper spray ionization (CFPSI)-MS, which allows a steady flow of internal standards (ciprofloxacin, Figure 1d) for semiquantitative analysis directly from biological tissue. We focused on rapid detection and in-depth characterization of the above drugs in solution, chicken breast tissue, and PDX models (Head and head and neck squamous cell carcinoma (HNSCC)) to better understand the origins of the observed spectral complexity in spray-based mass spectral analysis, to support unambiguous in laboratory medicine and quantitative analysis.

**Figure 1:**
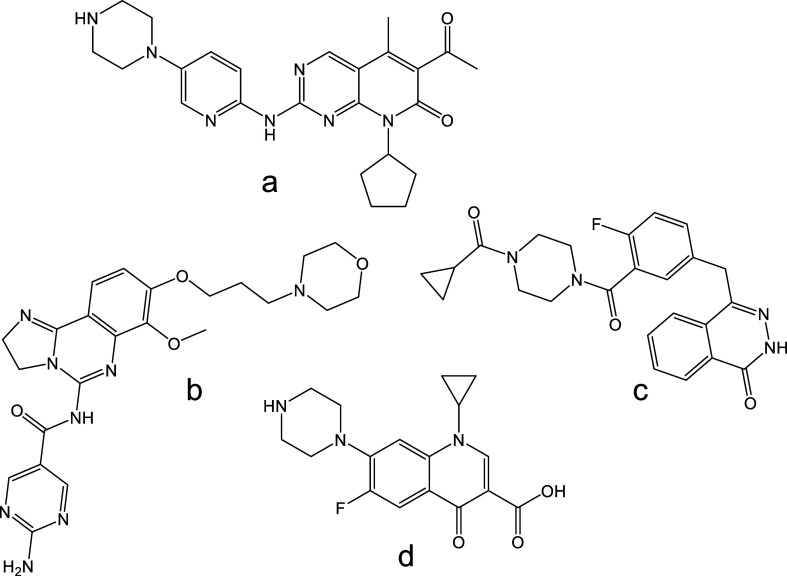
Chemical structures of the investigated anticancer drugs (a) palbociclib, (b) copanlisib, (c) olaparib, and (d) ciprofloxacin.

## Experimental Section

### Chemicals and Materials

Drug standards were purchased from Sigma-Aldrich (Steinheim, Germany), and aqueous standard solutions were prepared with Milli-Q water and used directly without further treatment. Chicken breast samples were obtained from a local grocery store. Whatman 42 filter papers were purchased from Sigma-Aldrich and were directly used without any pretreatment.

### PDX Tissue Sample Preparation

PDX model tissue samples were prepared by implanting a tumor tissue piece into mice, allowing for expansion. PDX models were generated at EPO Berli*n*-Buch GmbH (Berlin, Germany) and maintained through subcutaneous implantation in NMRI nu/nu or NOG mice (model HN15239; Janvier, France), following previously established protocols.^38^ In brief, the substances were administered via subcutaneous injections into the mice (PDX model). Doses and schedules were determined based on prior experience in animal experiments and represent the maximum tolerated or effective doses. The injection volume was 0.2 mL/20 g of body weight. Treatment was continued for 3 weeks, unless the tumor size exceeded 2 cm^3^ or the animals lost more than 10% of their body weight. EPO holds full accreditation from AAALAC. Tumor tissues were excised, sectioned into small fragments, and used for transplantation. The study received approval from the Institutional Review Board of Charité-Universitätsmedizin Berlin (EA4/019/12). All animal procedures complied with the UK Coordinating Committee on Cancer Research guidelines for animal welfare and the German Animal Welfare Act and were authorized by the relevant regulatory authority (LaGeSo Berlin, A0452/08).

### Reference Solutions and Chicken Tissue Sample Preparation

Reference solutions were prepared as 10 μM concentrations, unless otherwise mentioned.

A frozen chicken tissue sample was sliced into small pieces (∼1 mm^2^ area and ∼0.5 mm^2^ thickness). These samples were then individually dipped inside aqueous solutions of different concentrations. Samples were kept at 4 °C temperature overnight and then brought to room temperature for 30 min before being washed with milli-Q water to remove the residual drugs. The samples were placed close to the tip of the triangularly cut filter paper to perform paper spray. CFPSI MS was performed by passing an aqueous ciprofloxacin solution onto and across the tissue sample.

### Mass Spectrometry

Quantitative MS measurements were performed on a Thermo (Bremen, Germany) LTQ XL instrument with a modified ion source to accommodate CFPSI. For CFPSI MS, a triangular cut Whatman 42 filter paper (7 mm high, 10 mm base) was placed in front of the inlet of the mass spectrometer at a 5–8 mm distance by means of a copper clip connected to a high-voltage power supply (Figure 2). Subsequently, the tissue sample was placed on top of the paper close to the tip of the paper. A continuous source of internal standard (aqueous ciprofloxacin solution) was pumped onto the sample using a fused silica capillary, pushed from a syringe using a syringe pump at a flow rate of 5 μL/min. During all CFPSI MS experiments, the voltages for the tube lens and capillary were set to +35 and +110 V, respectively. The sheath gas pressure was zero and the capillary temperature was set to 270 °C. All CFPSI MS measurements were recorded for 2.5–2.8 min of acquisition time using the following instrument settings for the Thermo LTQ MS: mass range was set to normal, scan type was set to full, and microscans were set to 5, respectively. The first 0.5–0.8 min were considered as instrument equilibrium time and the subsequent 2 min data were used for analysis.

**Figure 2:**
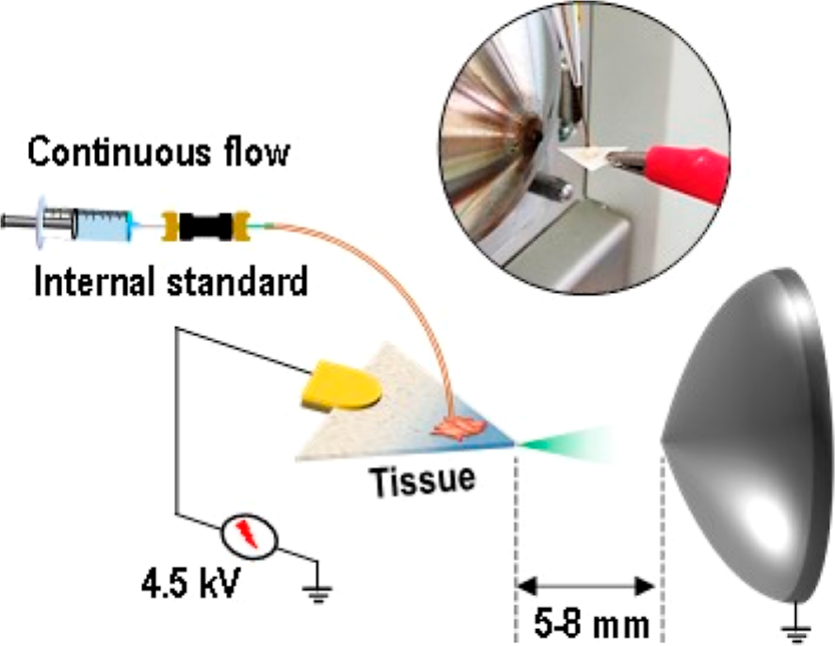
Schematic of CFPSI MS showing the process of tissue measurements with a continuous flow of the internal standard ciprofloxacin.

## Results and Discussion

### Characterization of CFPSI MS

During CFPSI MS, we observed a fine electrospray generated from the tip of the paper upon the application of +3 kV. We initially characterized the CFPSI MS setup using chicken breast tissue samples as a mimic of human tissues. We spiked the samples with standards of palbociclib, copanlisib, and olaparib at various concentrations to create a calibration curve. Note that an aqueous ciprofloxacin solution was used as an internal standard at a concentration of 5 μM, delivered in continuous flow. This resulted in protonated molecules [d + H]^+^ at *m*/*z* 332 in positive ion mode (refer to Supporting Information; Figures S1 and S2 illustrate the fragmentation pathway of ciprofloxacin^39^). A selected ion chronogram of the peak at *m*/*z* 332 is shown in Figure S3, displaying a stable signal intensity over time. In the tissue sample spiked with palbociclib, a signal was observed at *m*/*z* 448 along with *m*/*z* 332, indicating the presence of the protonated drug palbociclib [a + H]^+^ (Figure 3a). The signal stability was verified with an extracted ion chronogram, as shown in Figure S4. This was further confirmed by MS/MS analysis of the isolated species, which gave a major product ion at *m*/*z* 380 and a minor signal at *m*/*z* 405 in the collision-induced dissociation (CID) spectra (Figure S5). We also performed MS^3^ tandem MS experiments of these two species, which are summarized in the Supporting Information (Figure S6, along with a proposed fragmentation pathway of palbociclib, Figure S7). Interestingly, in the CID spectrum of *m*/*z* 448, we also noticed a very small signal at *m*/*z* 447, corresponding to the radical cation [a]^•+^, which upon CID resulted in a major peak at *m*/*z* 379 and a signal at *m*/*z* 405, respectively (for interested readers, these spectra are summarized in the Supporting Information, Figure S8).

**Figure 3:**
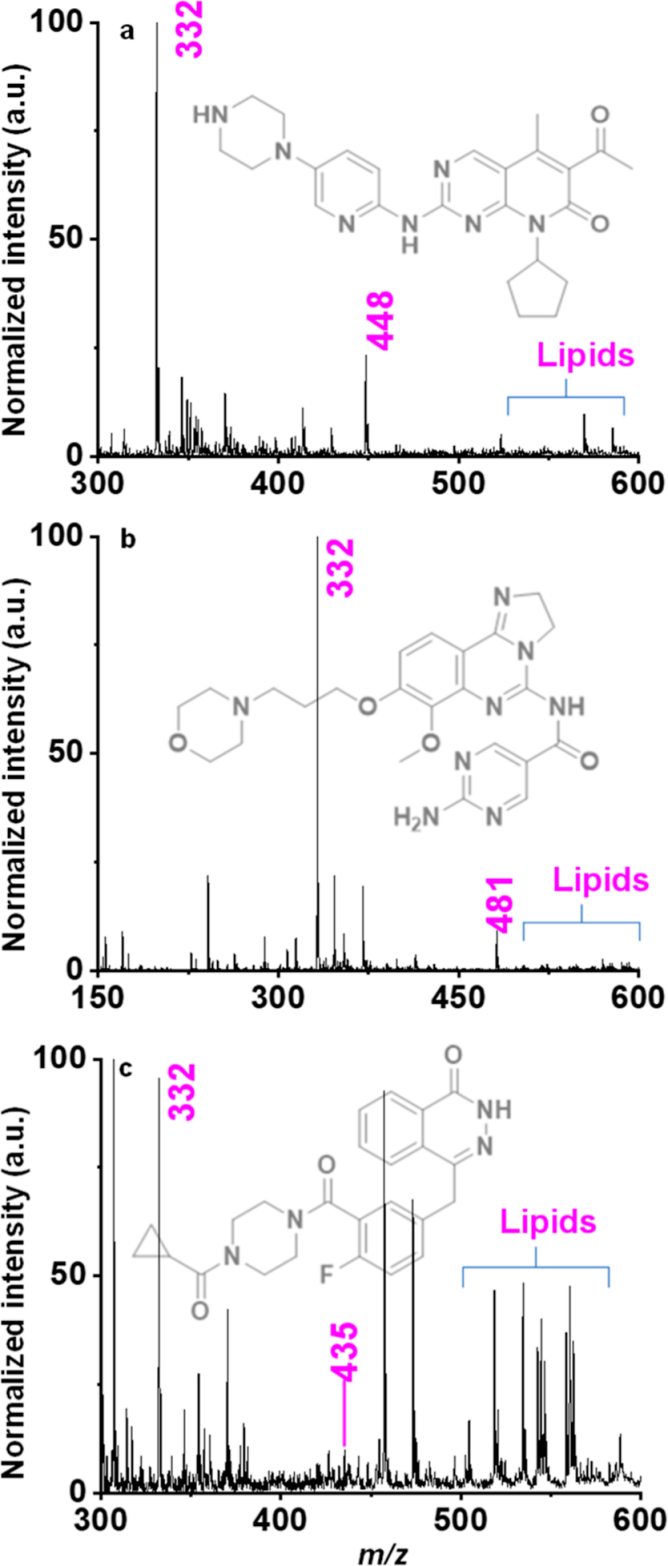
CFPSI MS of chicken breast tissue samples adsorbed with drugs at a 100 μM concentration, showing the protonated molecule peak of ciprofloxacin at *m*/*z* 332 (internal standard) and the protonated molecule peaks of (a) palbociclib at *m*/*z* 448, (b) copanlicib at *m*/*z* 481, and (c) olaparib at *m*/*z* 435.

Figure 3b,c displays the mass spectra observed for copanlisib [b + H]^+^ and olaparib [c + H]^+^, with fragmentation pathways proposed in the Supporting Information (Figures S9–S11). We also obtained phospholipid envelopes visible in the full scan mass spectra, which are present in the tissue. Upon analyzing the mass spectra presented in Figure 1, it was also observed that the relative signal intensities of copanlisib and olaparib are significantly lower than those of palbociclib. Samples prepared by spiking drugs at various concentrations also exhibited similar results (Figures S12–S14). We suggest that the observed low absorption levels of copanlisib and olaparib compared to palbociclib in the chicken tissue samples are attributed to specific physiological factors such as drug solubility, lipophilicity, membrane thickness, and surface area influencing their bioabsorption.

### In-Source Fragmentation

We found two additional peaks in the CFPSI MS spectrum of palbociclib in chicken tissue, at *m*/*z* 380 and 381, along with the protonated molecule signal for the drug molecule (Figure S15; expanded *m*/*z* region of the selected range for Figure 3a). The MS/MS spectrum of *m*/*z* 381 in Figure S16a displayed a distinct fragmentation pattern, primarily consisting of fragment ions at *m*/*z* 363, 338, 320, and 299. MS/MS analysis of *m*/*z* 380 exhibited a similar fragmentation pattern, but peaks were consistently one *m*/*z* unit lower than those observed in the spectrum for *m*/*z* 381 (Figure S16b). Note that the intensity ratio of these two peaks varied dramatically depending on the sampling method (see the discussion below). This indicates that the *m*/*z* 381 peak is not an isotope of *m*/*z* 380. We expected the MS/MS spectrum of *m*/*z* 380 to closely resemble the MS^3^ spectrum of the *m*/*z* 380 fragment ion, which originated from the *m*/*z* 448 precursor ion as presented in Figure S4. We suggest that these two ions are the result of the ISF of the protonated molecule of palbociclib. In Figure S17, we present a possible pathway for such an ISF reaction and the fragmentation by CID.

### Effects of Solvents and Additives

The selection of the solvent plays a critical role in MS, as ionization efficiencies can differ significantly depending on the polarity and ionizability of the solvent used. Often, organic solvents are used as eluents and organic acids (e.g., formic acid) are added to enhance ionization. We performed CFPSI MS with methanol, a mixture of methanol and formic acid, and acetonitrile. For the first two solvent systems, we observed in-source derivatization of palbociclib, as evidenced by the appearance of two new peaks at *m*/*z* 478 and 492, along with the protonated molecule peak of the drug (Figure 4a,b). Based on the fragmentation pattern of these signals (Figure 4d,e) and the observed required high collision voltages (30–40 V), we propose that the drug has undergone a nucleophilic addition reaction with methanol and formic acid. A possible reaction scheme and fragmentation pathway for these ions by CID are presented in Figures S18 and 19.

**Figure 4:**
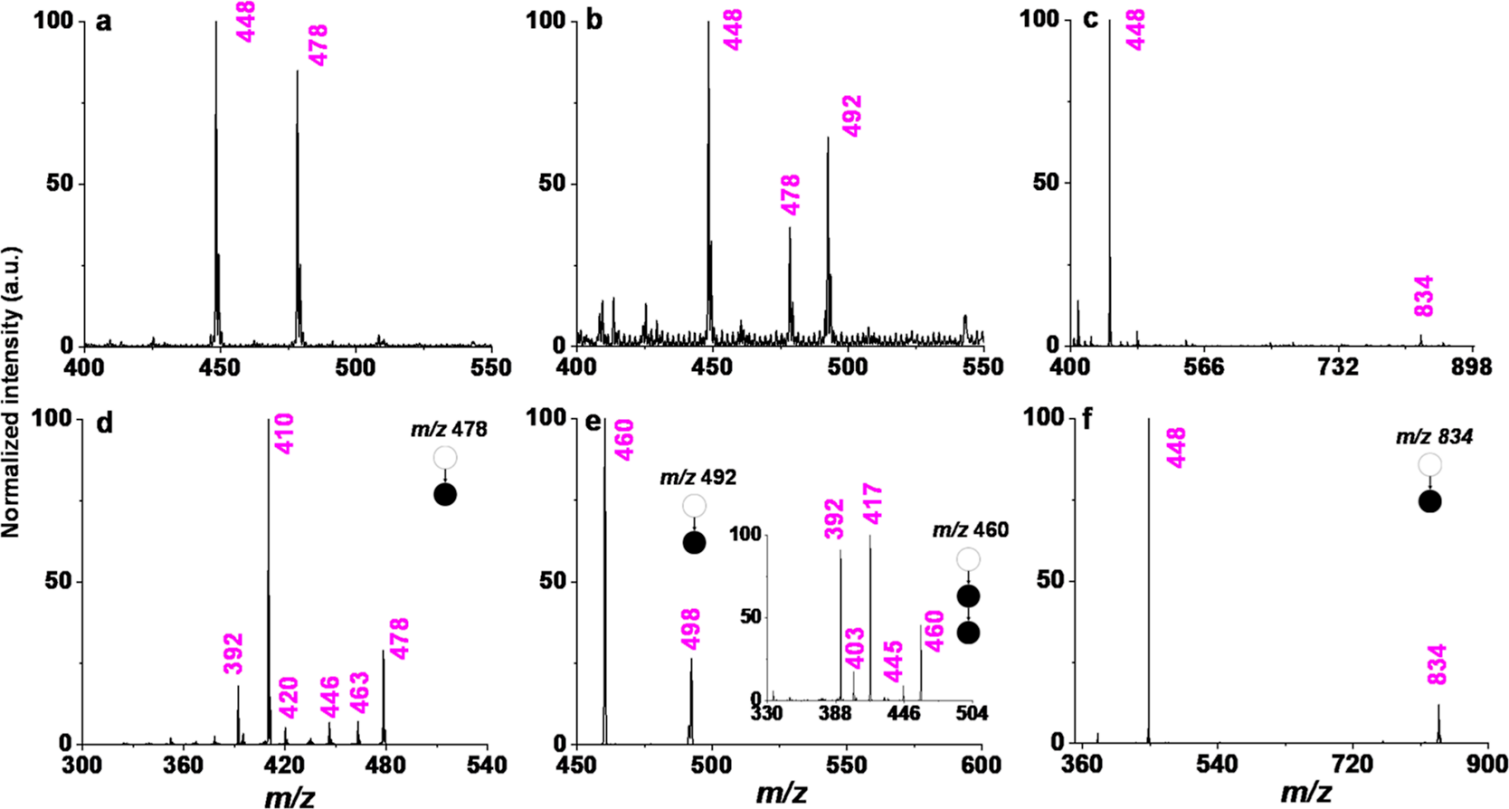
In-source chemical derivatization of analytes. (a–c) MS of palbociclib in methanol, formic acid-added methanol and acetonitrile; (d–f) tandem MS of isolated peaks at *m*/*z* 478, 492 and 834, respectively.

On the other hand, when we used acetonitrile as the solvent, we found an additional peak at *m*/*z* 834 (Figure 4c). MS/MS analysis of this ion suggests that it is an ion cluster of the protonated molecule of palbociclib and an ISF ion (Figure 4f). The proposed reaction mechanism is presented in Figure S20. We did not observe this peak in the two polar protic solvents.

We observed that the polarity of the solvent influenced the ISF of palbociclib, as demonstrated by experiments where a reference solution of palbociclib in various solvents was continuously delivered into the MS. During these experiments, we noticed that measuring pure solutions yielded only the ion at *m*/*z* 381. While both water and methanol contributed to ISF, this ion was completely absent when acetonitrile was used. This observation suggests that the ISF was assisted by the ionizability of the solvent. We further tested this hypothesis by adding different amounts of formic acid to the methanolic solution of the drug, discovering that a higher concentration led to even more fragmentation. Next, we prepared an aqueous solution of the drug with 10 μM of sodium carbonate to assess the effect of external ions. We observed a weak signal for the protonated drug molecule at *m*/*z* 448, while the predominant ion was the fragment at *m*/*z* 381. The relative abundance of *m*/*z* 381 with respect to the protonated molecule of the drug at *m*/*z* 448 in different solutions is presented in Figure 5. We hypothesize that this extensive fragmentation occurs as a result of ionic interactions during ionization. However, a detailed investigation of the exact mechanism of this fragmentation is beyond the scope of the current study.

**Figure 5:**
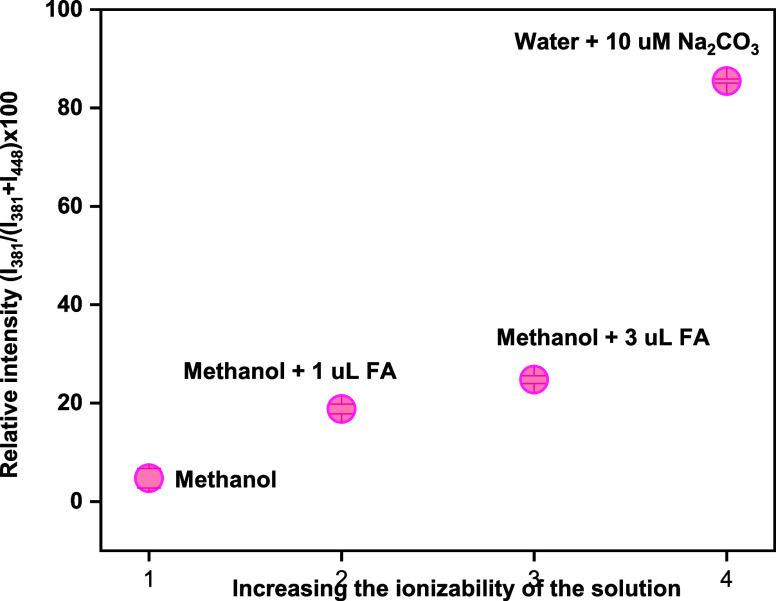
Conversion ratio of *m*/*z* 381, with increasing ionizability in solution.

### Calibration

Using different concentrations of the drugs spiked in chicken breast tissue, we evaluated the dynamic range of analysis in the tissue. To assess the reproducibility of CFPSI MS measurements, we calculated the coefficient of variation (CV %) from three replicate measurements from different portions of the same tissue, each spiked with palbociclib on separate days. The signal intensities were found to be invariable with time (Figure S21), showing a CV % of 9.4, 9.3, and 7.0%, respectively. The spatial variabilities were also found out to be minimal, as the estimated CV % calculated from different tissue portions is 17.5%. The calibration curve exhibited nonlinearity, saturating at higher concentrations (Figure 6). Such nonlinearity in the calibration curve is often caused by matrix effects, saturation during ionization, dimer or multimer formation, isotopic effects, and detector saturation in traditional LC–MS experiments.^40–42^ We believe that the nonlinear nature of the calibration curve in our measurements is due to the retention of the drugs in the tissue samples at high concentrations during elution. We speculate that the tissue retains a certain amount of drug molecules at significantly high concentrations and releases them only slowly.

**Figure 6:**
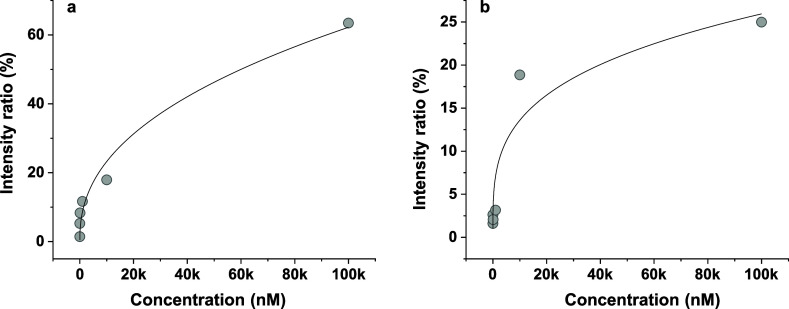
Calibration curves of (a) palbociclib and (b) copanlisib using CFPSI MS. The nonlinearity is due to matrix effects.

### CFPSI MS of PDX Samples

Next, we conducted an analysis of the PDX samples using CFPSI MS. Figures S22–S27 illustrate the mass spectra of six tissue samples. Each PDX model was treated with three distinct chemotherapeutic agents and categorized based on similar medical treatments. Interestingly, our findings revealed variations in the signal intensities of the drugs, suggesting that further investigation could provide valuable insights into the differential responses of the models. For example, sample 3F highlights the most significant absorption of palbociclib in the tissue, as evidenced by the prominent protonated molecule ion peak at *m*/*z* 448 (Figure S24). The detectable concentration using CFPSI MS was found to be approximately 132 μM in sample 3F. While we found the lowest absorption for copanlisib among the samples, olaparib is completely absent in any of these samples. This suggests that drug absorption can vary from patient to patient and may depend on physiological conditions.

### Co-Relation between the Chicken Breast and PDX Model Tissue Samples

We compared the mass spectra between PDX and chicken breast tissue to reveal any differences between the investigated drugs upon absorption. While we did not observe major differences for ciprofloxacin, we found that palbociclib underwent ISF leading to *m*/*z* 380. In Figure 7, we present comparative mass spectra collected from the reference 100 μM solution of palbociclib and chicken tissue spiked with 100 μM aqueous palbociclib, and PSX sample 3F. Based on our observation from pure solution and PDX tissue samples above, we suggest that *m*/*z* 380 is formed in the tissue sample and not during ionization. Such a biotransformation can be the result of several factors in tissue including change in pH, temperature or enzymatic action leading to hydrolysis, redox reactions and degradation of the drug.^43^

**Figure 7:**
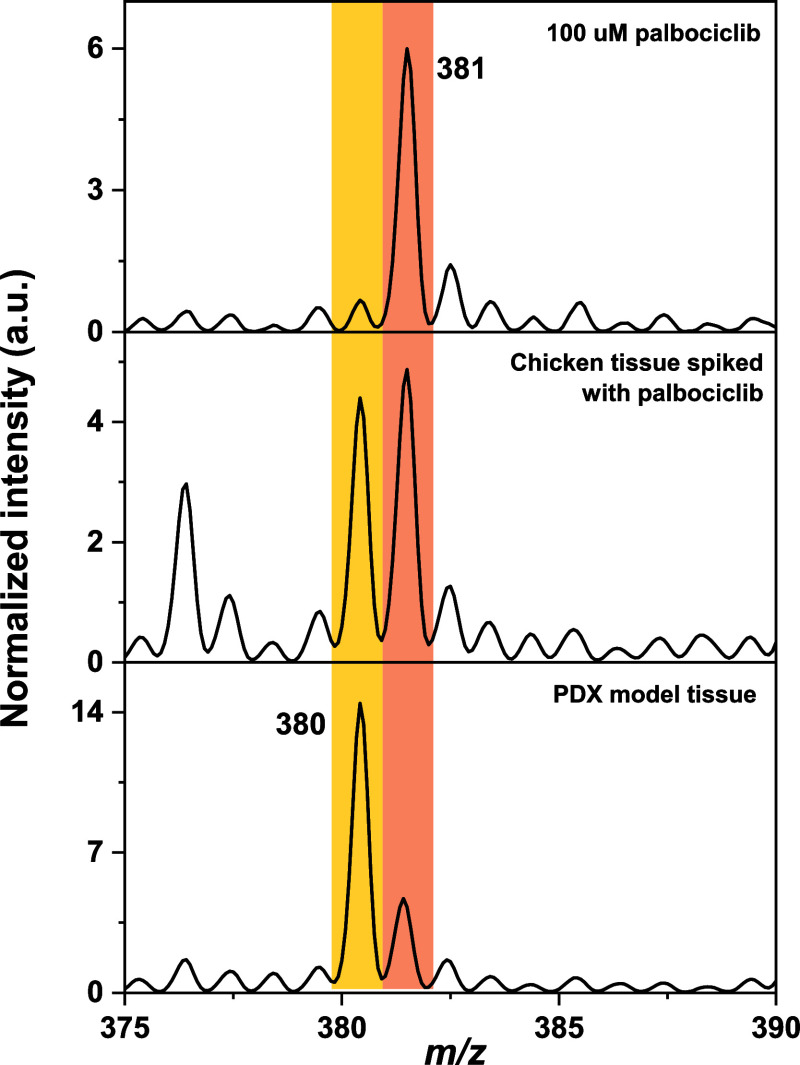
Comparative mass spectra of ISF peaks of palbociclib in different samples. Yellow and orange traces indicate two in-source fragmented species (i.e., *m*/*z* 380 and 381).

## Conclusion

In summary, we have successfully demonstrated the application of continuous flow paper spray ionization mass spectrometry (CFPSI MS) to the analysis of anticancer drugs from tissue samples. This innovative technique employed a continuous flow of internal standards, enabling semiquantitative analysis of the drugs from the biological tissue samples. The approach is much faster than conventional LC–MS assays and also allowed in-depth characterization of the investigated drugs in complex biological matrices. The results demonstrated the complexity of the mass spectra, as a result of ISF, solvent–solute reactions, and ion clustering. The underlying chemistry was also influenced by the solvent composition, ionizabillity of the solvent, and presence of ionic species such as acid, bases, and salts. Our recent findings on protomer formation, combined with the current results, suggest that even within a single component system, mass spectra can exhibit considerable complexity. Therefore, it is essential to exercise caution when assigning and quantifying species in conventional MS measurements.

Our methodology broadens the scope of the ambient PSI techniques. The method was applied to PDX model tissue samples to understand the absorption of selected anticancer drugs. Using CFPSI MS, we found differential absorption of drugs in different tissues, which were treated similarly. We believe that our findings will support the development of future rapid quantitative CFPSI assays for tissue samples.

## Supplementary Material



## Notes

Sampling methods, mass spectrometric sample preparation, details of setting up CFPSI MS, full-range mass spectra, MS/MS spectra, IMS spectra, and the fragmentation pathway of these drugs (PDF)
