# Supplementary material for: Continuous Flow Paper Spray Ionization Mass Spectrometry for In-Depth Characterization of Anticancer Drugs in Tissues: Addressing Mass Spectral Complexity
Source: J Am Soc Mass Spectrom. 2026 Jan 16;37(2):483–90. doi: 10.1021/jasms.5c00374 (PMC12879924; doi:10.1021/jasms.5c00374)
Supplement: Supplementary file 1 [file js5c00374_si_001.pdf]

Supplementary Information

Continuous flow paper spray ionization mass spectrometry for in-depth characterization of anticancer drugs in tissues: addressing mass spectral complexity

Pallab Basuri,[a] Konrad Klinghammer,[b] Oliver Klein,[c] and Dietrich A. Volmer[*a]

a Department of Bioanalytical Chemistry, Institute of Chemistry, Humboldt-Universität zu Berlin, 12489 Berlin, Germany.

b Hematology, Oncology and Tumor Immunology, Charité - Universitätsmedizin, 12200 Berlin, Germany.

c Center for Regenerative Therapies, Core Unit Imaging Mass Spectrometry, Charité - Universitätsmedizin, 13353 Berlin, Germany

*Corresponding author:

Prof. Dr. Dietrich Volmer

Humboldt University Berlin

Department of Bioanalytical Chemistry

12489 Berlin, Germany

Tel +49 30 2093 7588

Email: Dietrich.Volmer@hu-berlin.de

Table of contents

Description **Page No.**

Figure S1: MS and MSMS or ciprofloxacin ……………………………………………………..…….3

Figure S2: Fragmentation pathways of ciprofloxacin ……………………………………….……..4

Figure S3: Extracted ion chronogram for the molecular ion of the internal standard …………….5

Figure S4: Extracted ion chronogram for the molecular ion of palbociclib …………………………6

Figure S5: MS/MS spectrum of the isolated species at *m/z* 448 ……………………………………7

Figure S6: MS^3 of isolated peak at m/z 380 and 405, respectively ……………….……………….……8

Figure S7: Fragmentation pathways of pablociclib …………………………………………….……..9

Figure S8: MSMS of isolated peak at m/z 447 ………………………………………………..…10

Figure S9: MS/MS spectra of copanlisib and olaparib ….……………………………………..…11

Figure S10: Fragmentation pathways of copanlisib …………………….………………….…12

Figure S11: Fragmentation pathways of Olaparib ……………………………………………………13

Figure S12: CFPSI MS of chicken tissue sample spiked with pablociclib ..……………….……….…14

Figure S13: CFPSI MS of chicken tissue sample spiked with colanlisib ..………………………..…15

Figure S14: CFPSI MS of chicken tissue sample spiked with olaparib .…………….……….…..…16

Figure S15: Selected mass range spectrum of Figure 3a……………….……………………………17

Figure S16: MS/MS spectrum of *m/z* 380 and 381……………………………………………………..18

Figure S17: Formation pathway of ion at *m/z* 381…………………….……………………………..19

Figure S18: Formation and fragmentation pathway of ion at *m/z* 478…………………………….20

Figure S19: Formation and fragmentation pathway of ion at *m/z* 492…………………………….21

Figure S20: Formation of ion at *m/z* 834………………………………………….………………..22

Figure S21: Comparative extracted ion chromatograms from replicate measurements …..……23

Figure S22-27: CFSI MS of PDX model tissue samples …………………………………..…24-29

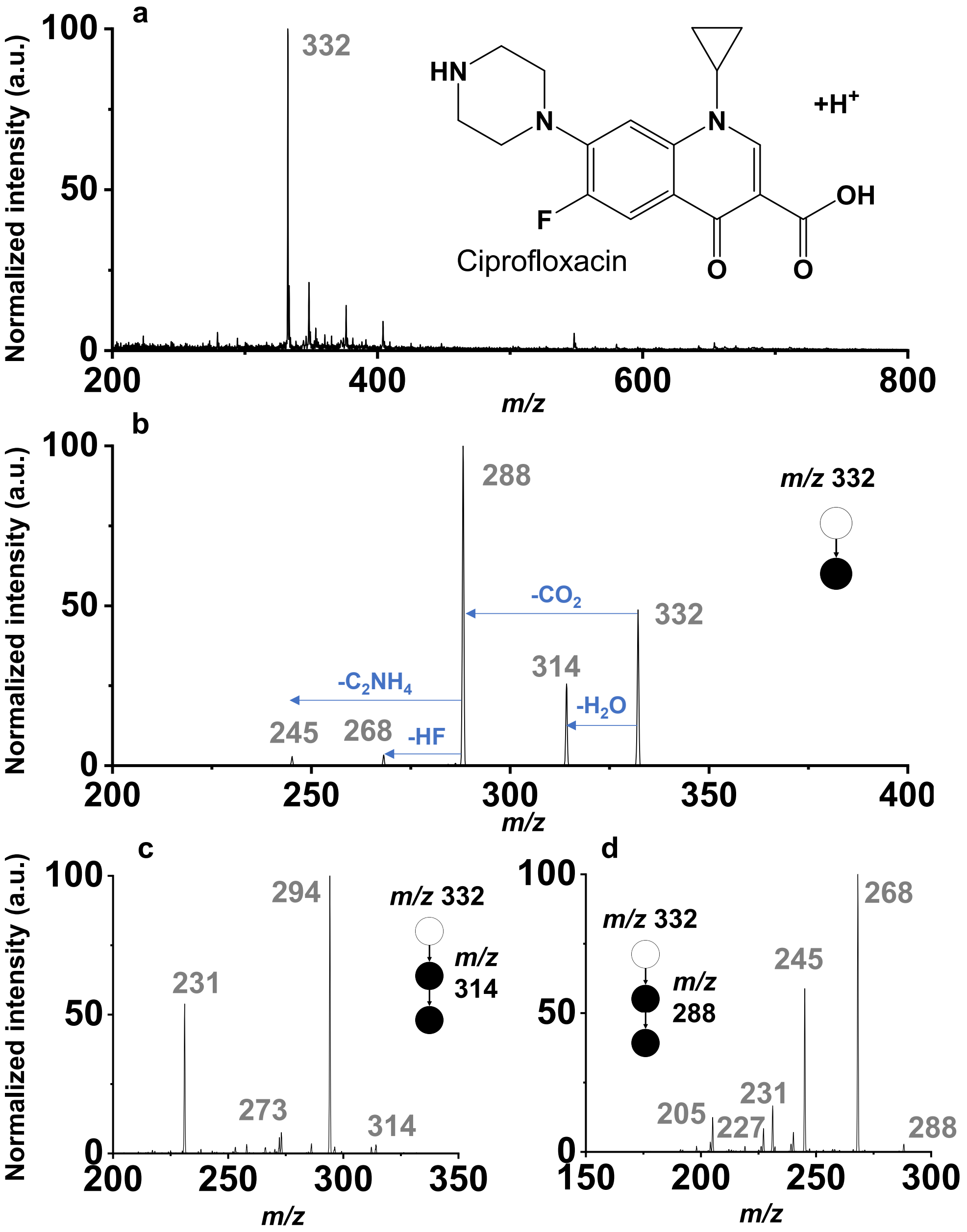

Figure S1. CFPSI MS of 5 uM aqueous ciprofloxacin. a) Full range mass spectrum of the drug. b) MSMS of the isolated peak at *m/z* 332. C and d) MS^3 of the isolated peak at *m/z* 314 and 288, respectively.

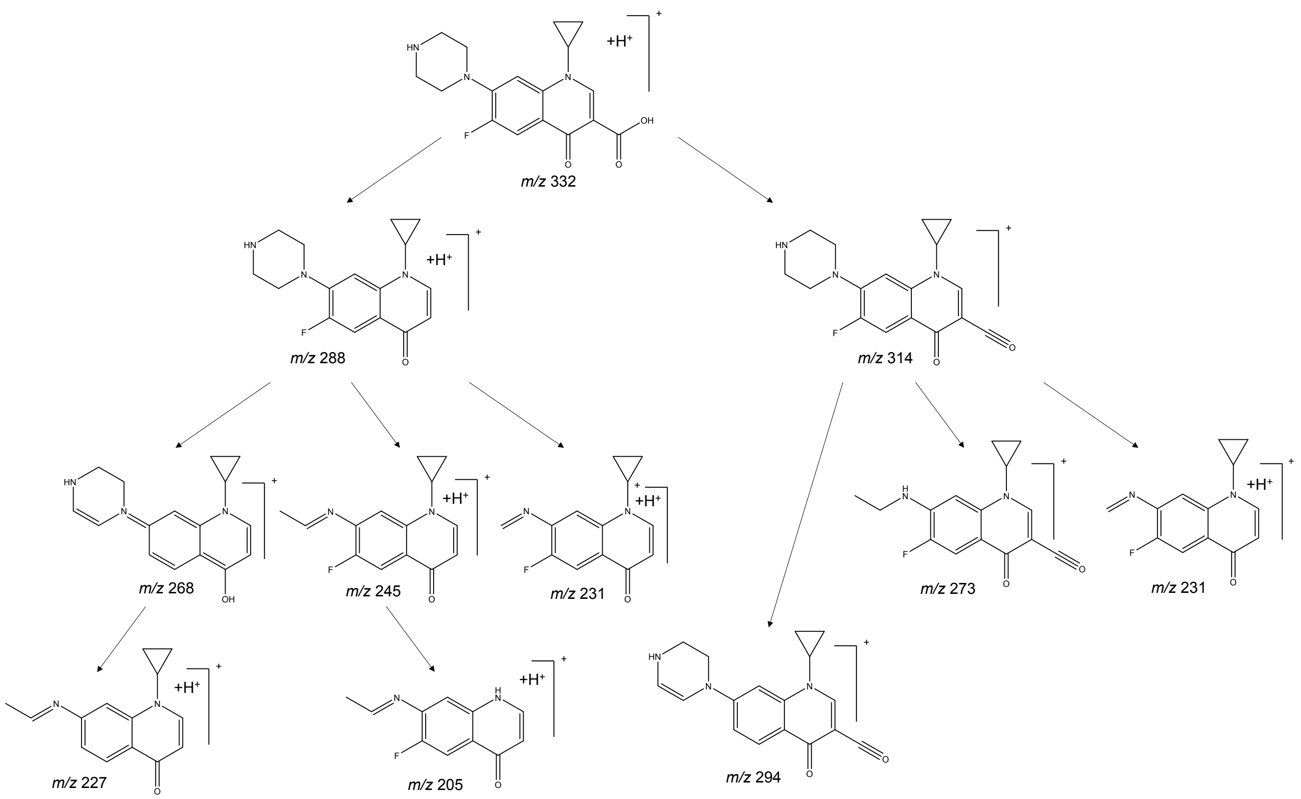

Figure S2. A proposed MS fragmentation pathway of ciprofloxacin based on the CID spectrum of the isolated molecular ion peak at *m/z* 332.

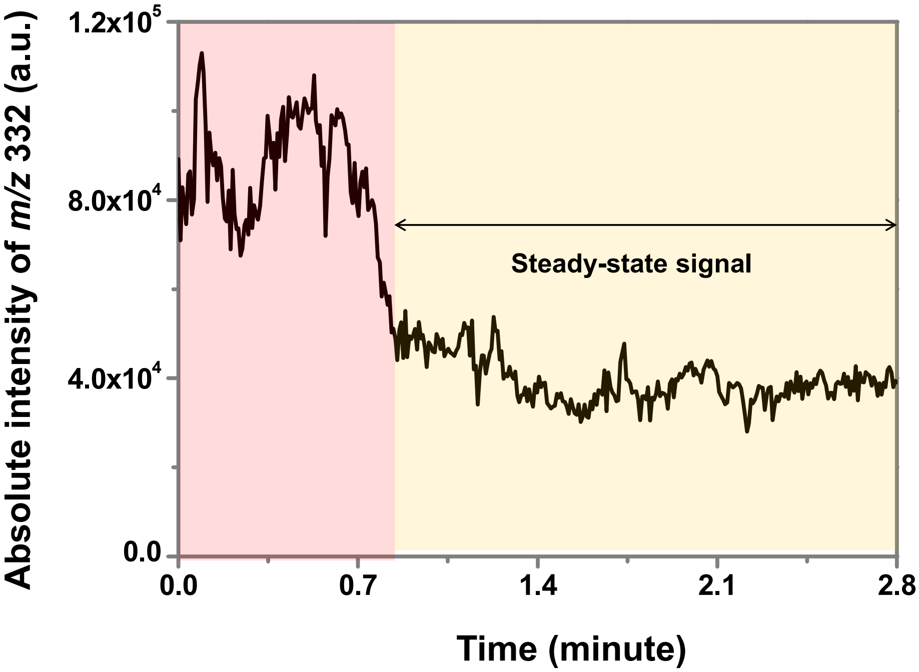

Figure S3. Extracted ion chronogram for the protonated molecule of the internal standard, ciprofloxacin at *m/z* 332 from the CFPSI MS measurement of chicken tissue samples spiked with palbociclib. The red area indicates the CFPSI equilibrium time, while the yellow area represents the data acquired for all mass spectrometry measurements

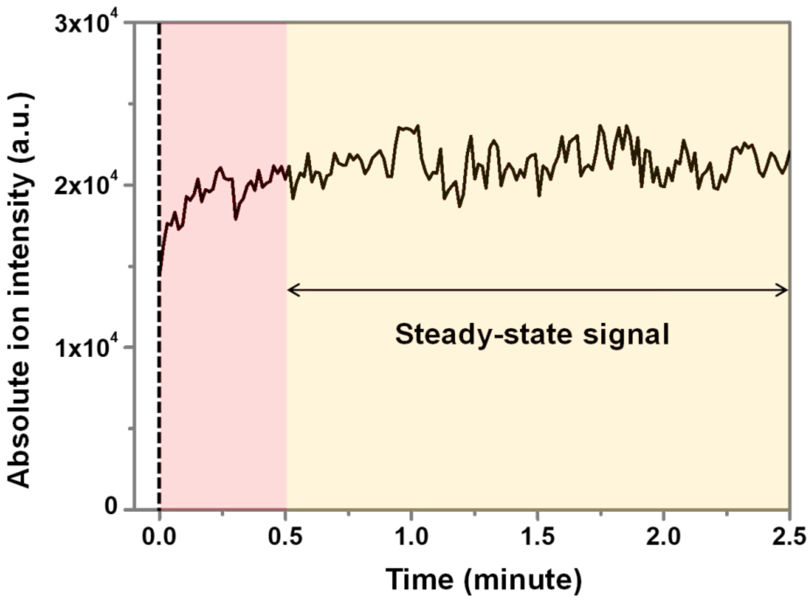

Figure S4. Extracted ion chronogram from the CFPSI MS measurement of chicken tissue samples spiked with palbociclib. The red area indicates the CFPSI equilibrium time, while the yellow area represents the data acquired for all mass spectrometry measurements.

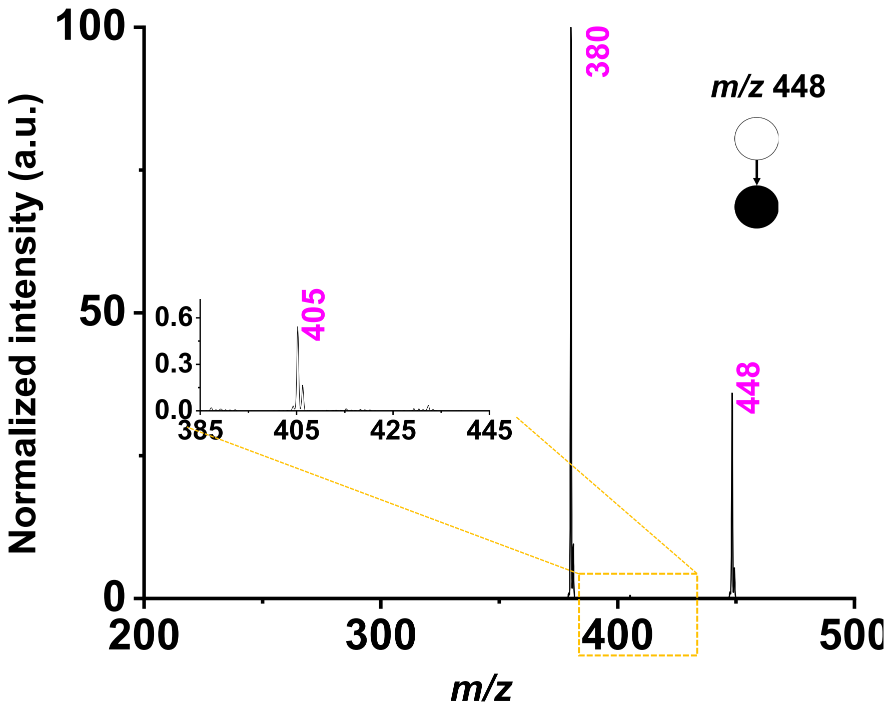

Figure S5. MS/MS spectrum of the isolated species at *m/z* 448, corresponding to protonated palbociclib.

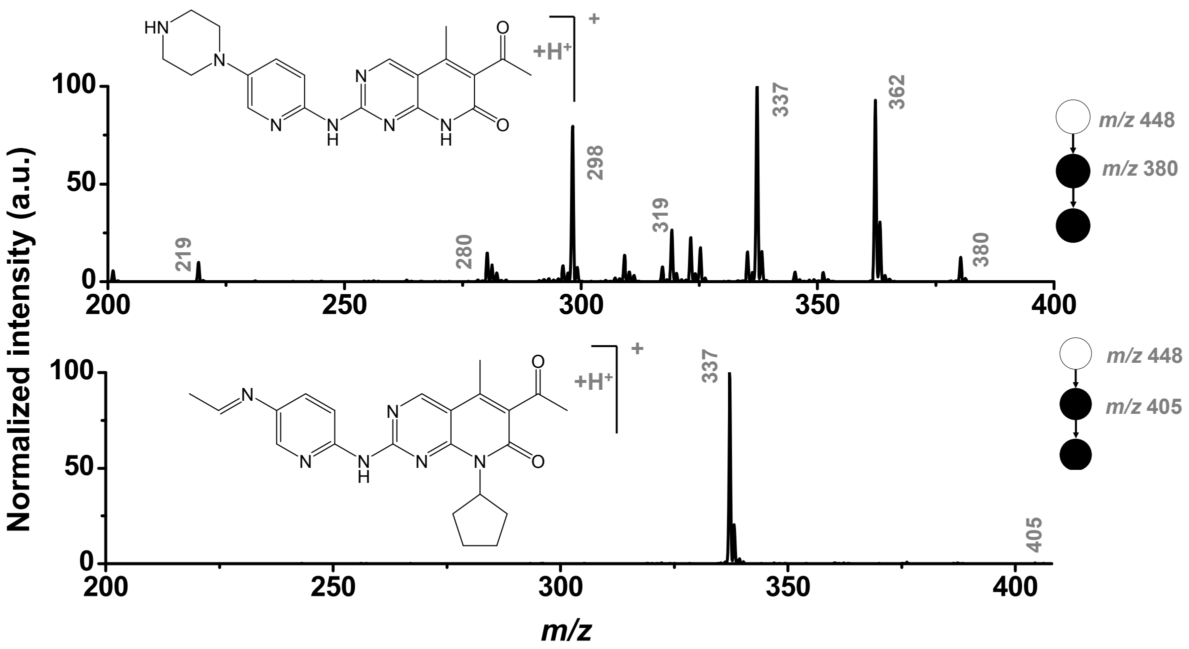

Figure S6. MS^3 of isolated peaks of *m/z* 380 (top) and 405 (bottom).

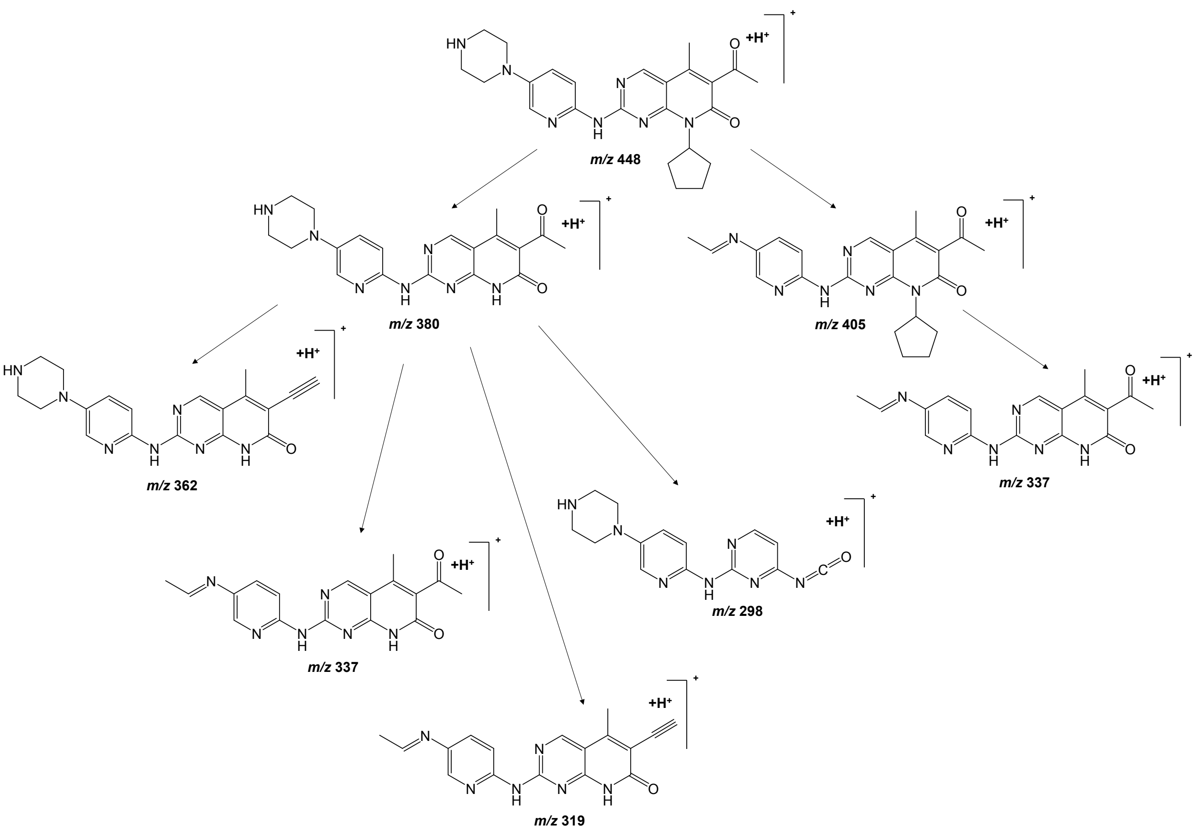

Figure S7. A proposed MS fragmentation pathway of palbociclib based on the CID spectrum of the isolated molecular ion peak at *m/z* 448.

a

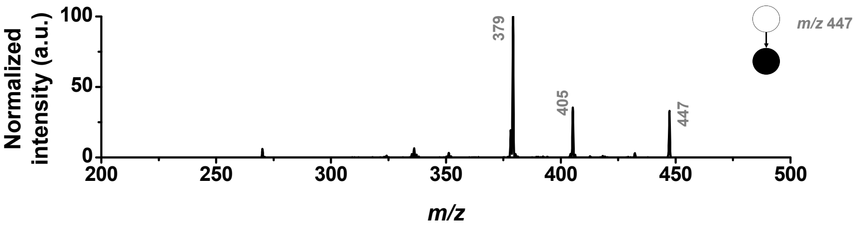

b

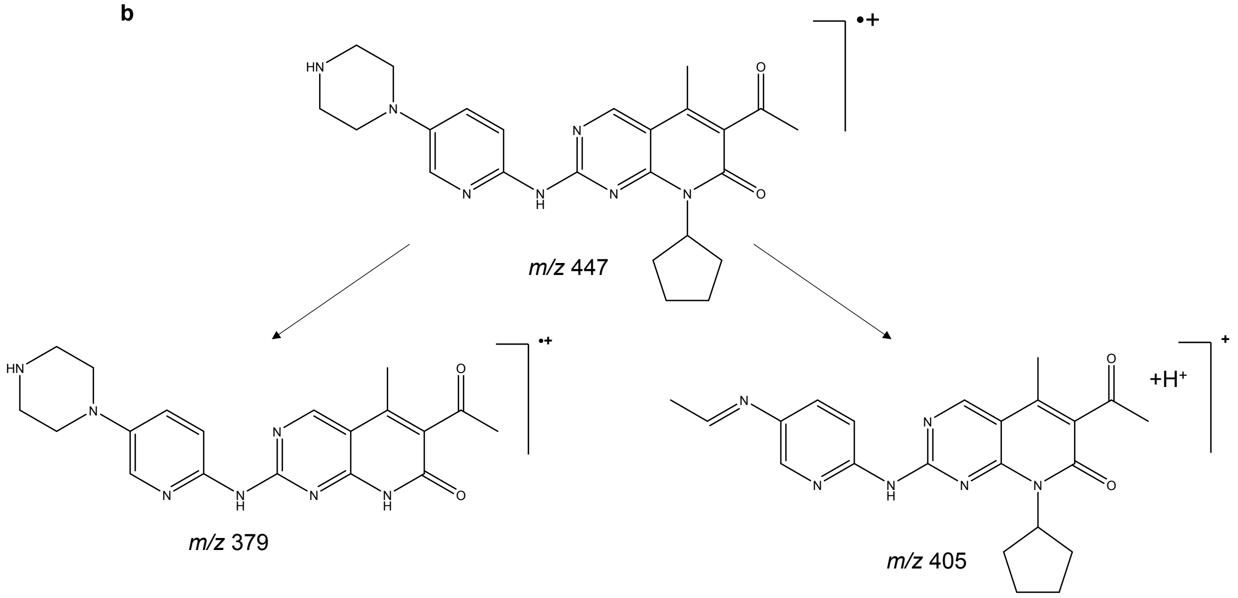

Figure S8. A) MS/MS spectrum of the isolated peak at *m/z* 447. B) Proposed MS fragmentation pathway of palbociclib based on the CID spectrum of the isolated molecular ion peak at *m/z* 447 corresponding to the radical cationic species of the drug.

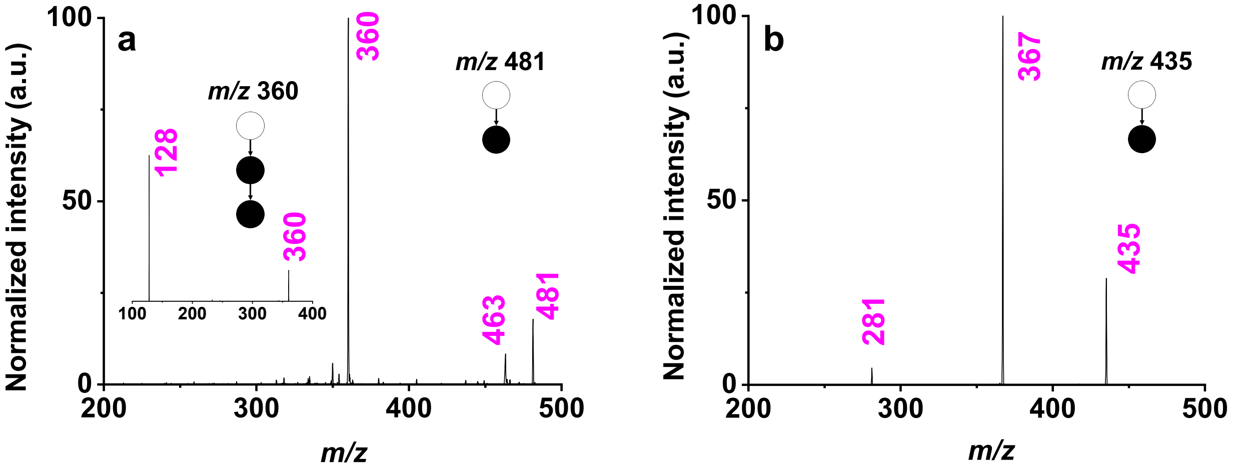

Figure S9. MS/MS spectra of the mass-selected isolated peaks of a) *m/z* 481 and b) *m/z* 435, correspond to molecular ion peaks of copanlisib and olaparib.

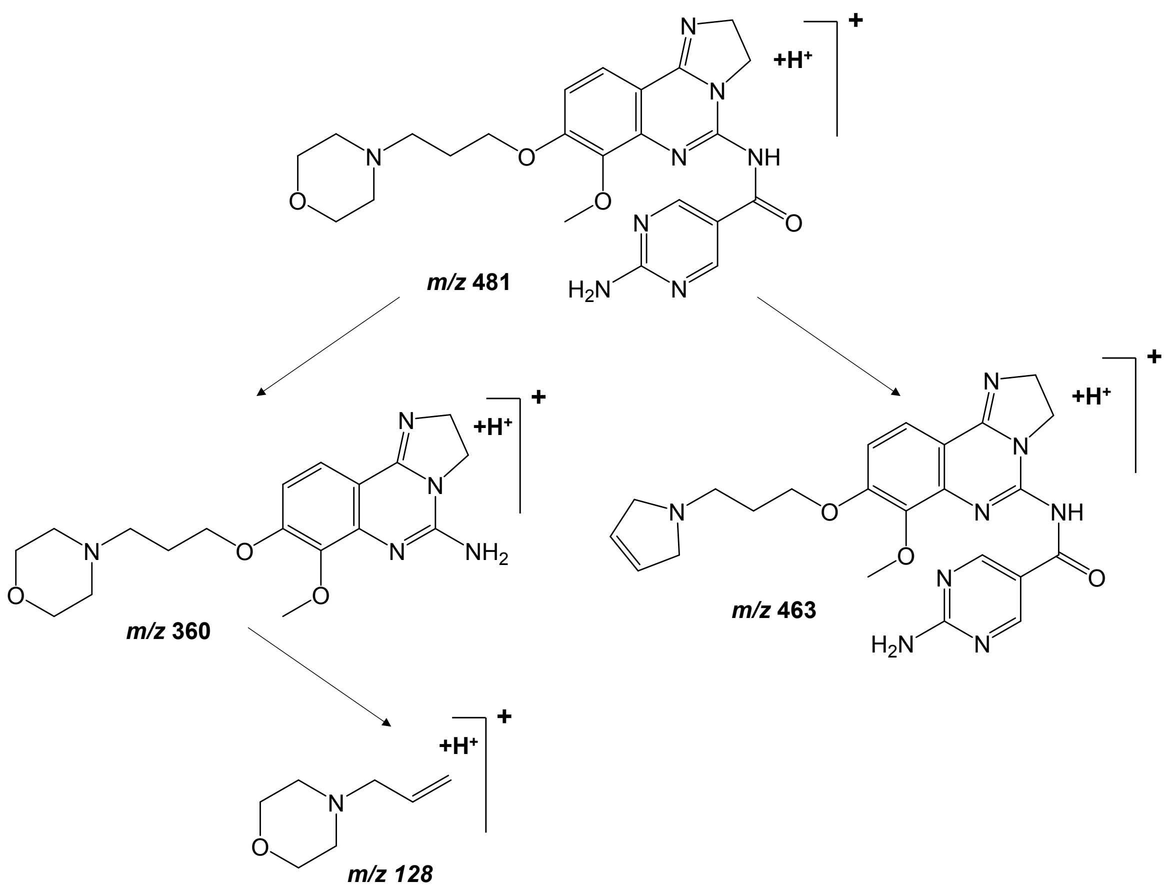

Figure S10. A proposed MS fragmentation pathway of copanlisib based on the CID spectrum of the isolated molecular ion peak at *m/z* 481.

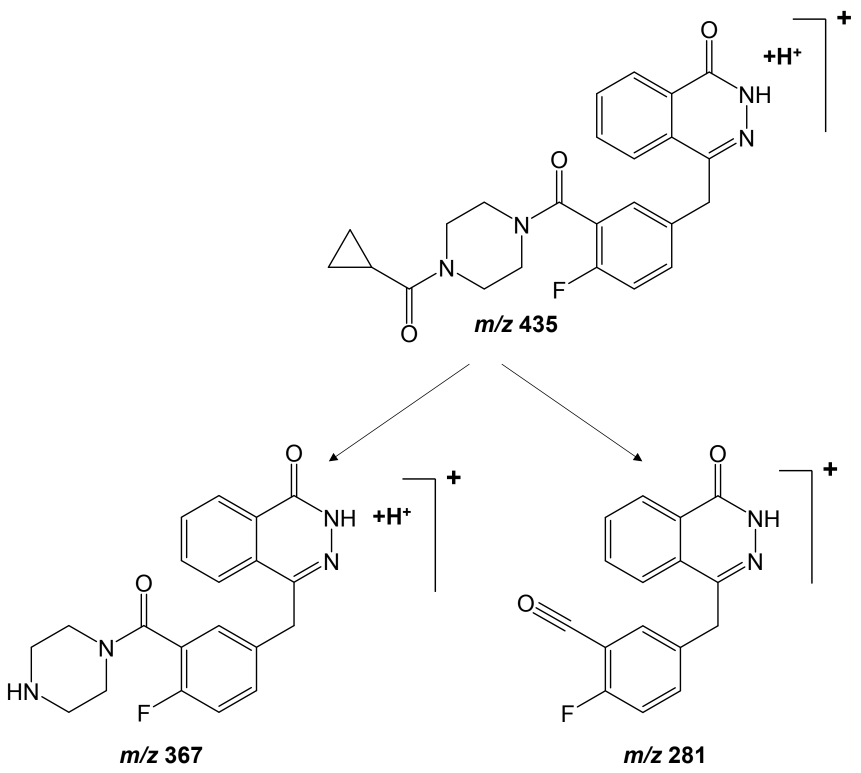

Figure S11. A proposed MS fragmentation pathway of olaparib based on the CID spectrum of the isolated molecular ion peak at *m/z* 435.

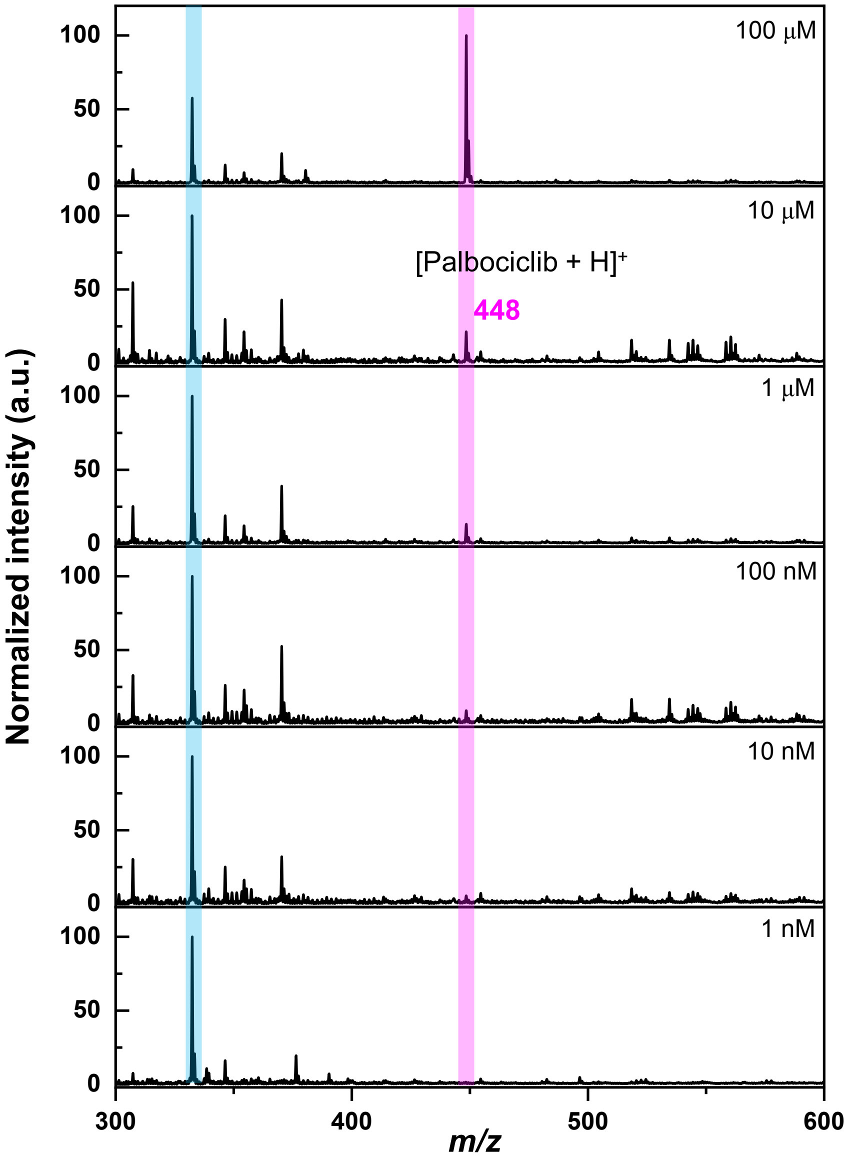

Figure S12. CFPSI MS of chicken breast tissue samples spiked with palbociclib at different concentrations ranging from 1 nM to 100 uM.

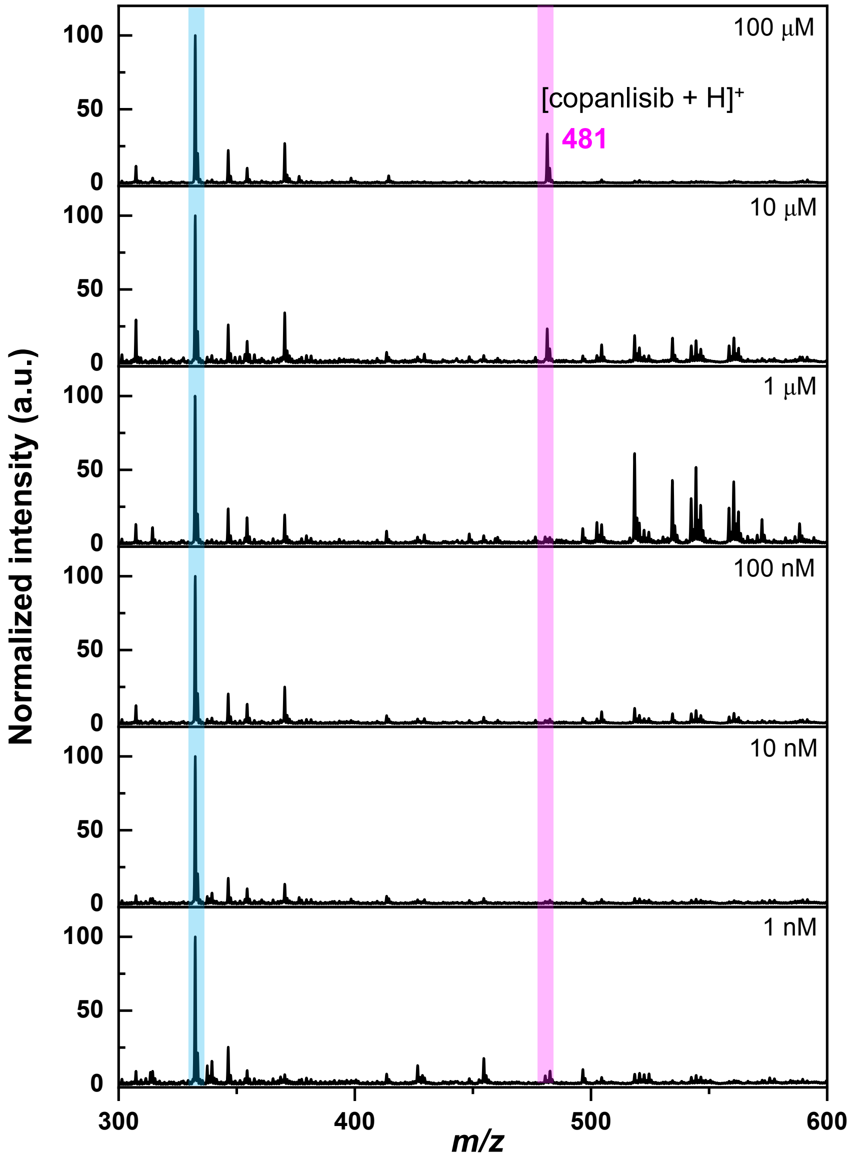

Figure S13. CFPSI MS of chicken breast tissue samples spiked with copanlisib at different concentrations ranging from 1 nM to 100 uM.

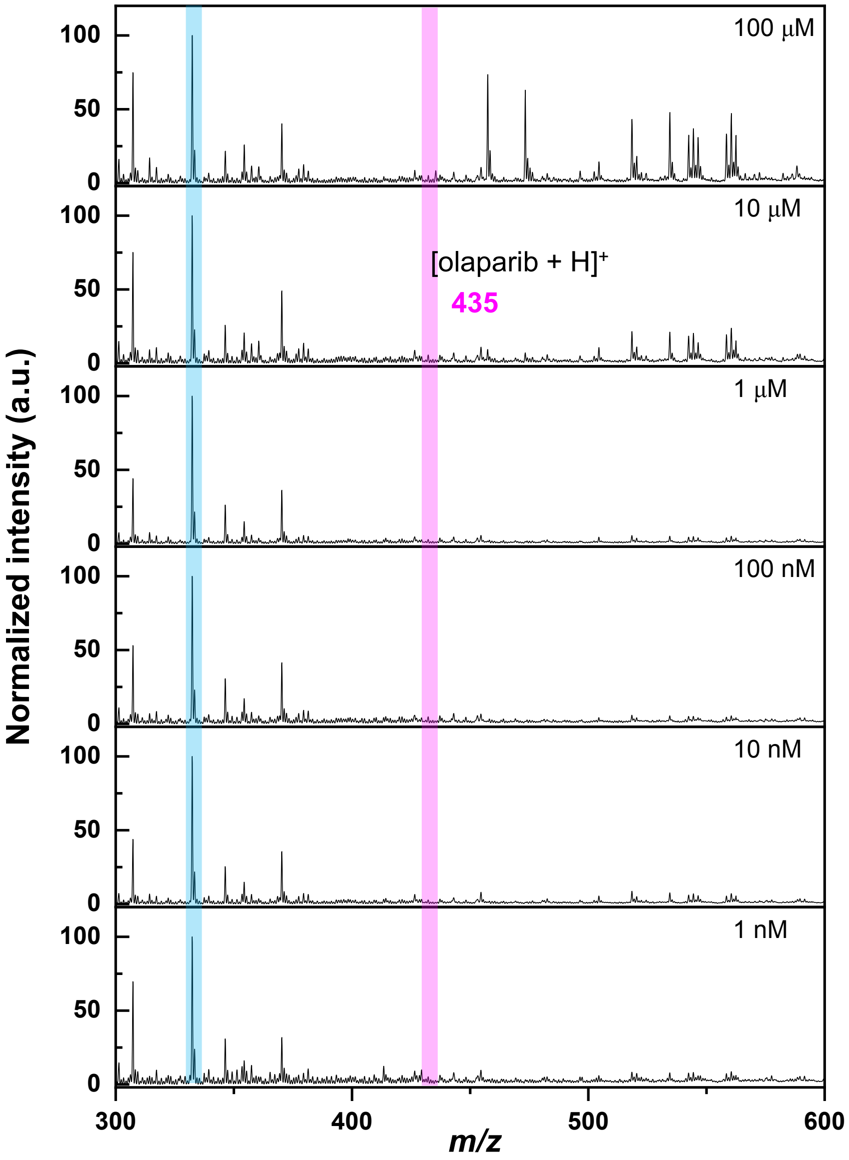

Figure S14. CFPSI MS of chicken breast tissue samples spiked with palbociclib at different concentrations ranging from 1 nM to 100 uM. Interestingly, we did not observe molecular ion peaks for Olaparib, even at higher concentrations.

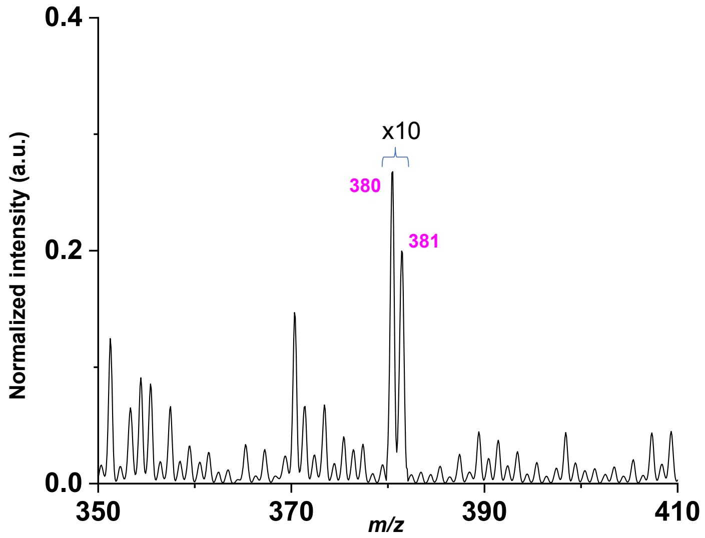

Figure S15. Selected mass range spectrum collected from a chicken tissue spiked with palbociclib (Zoomed in on Figure 3a).

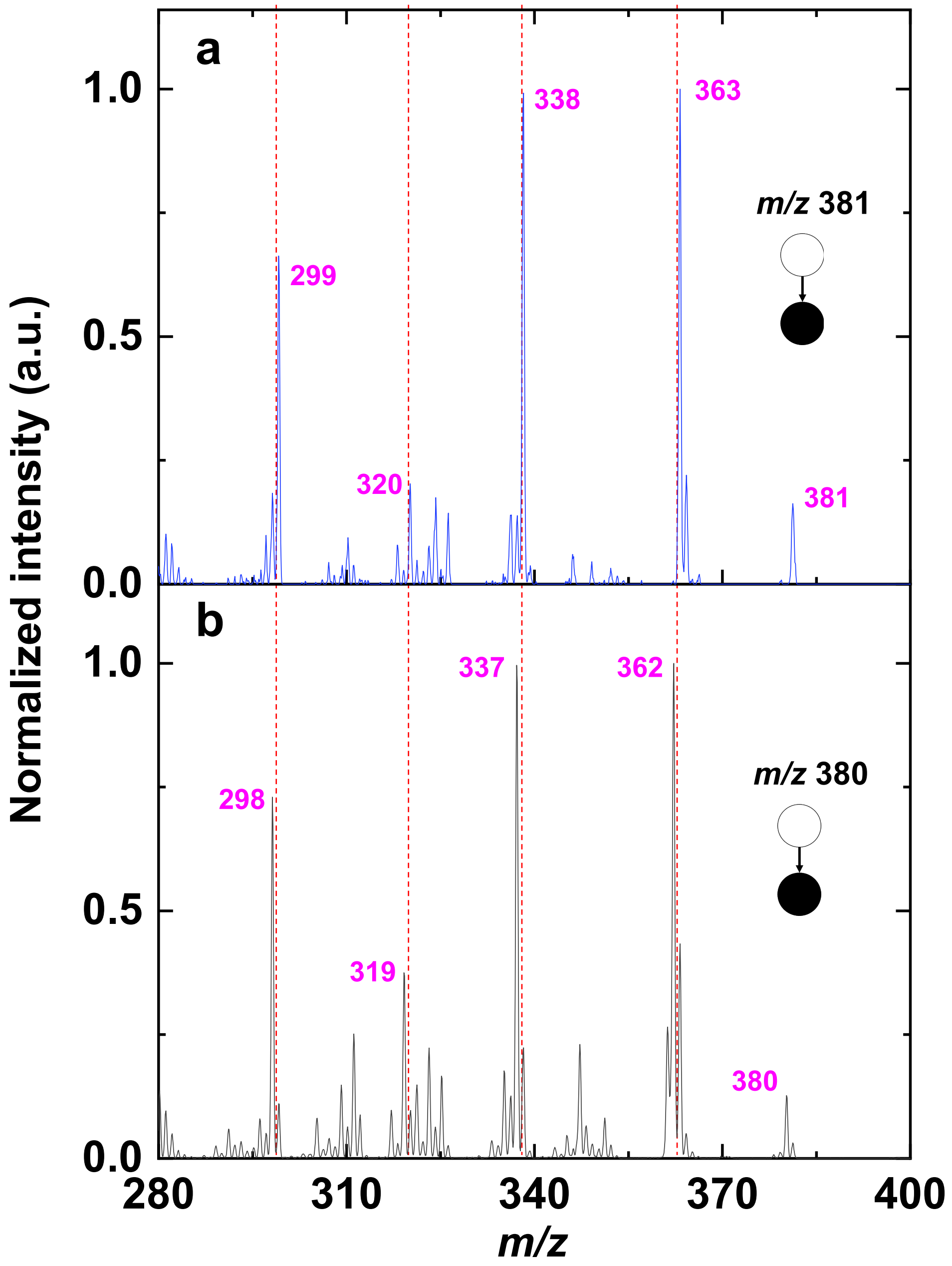

Figure S16: MS/MS spectrum of a) *m/z* 381 and b) *m/z* 380, respectively.

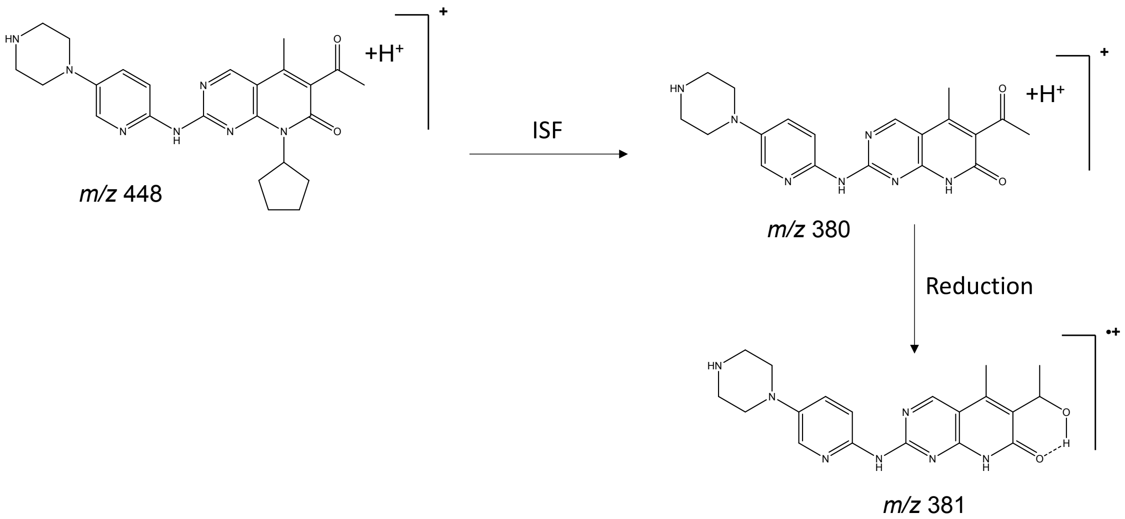

Figure S17: Formation pathway of the ISF species detected for palbociclib at *m/z* 380 and 381, respectively.

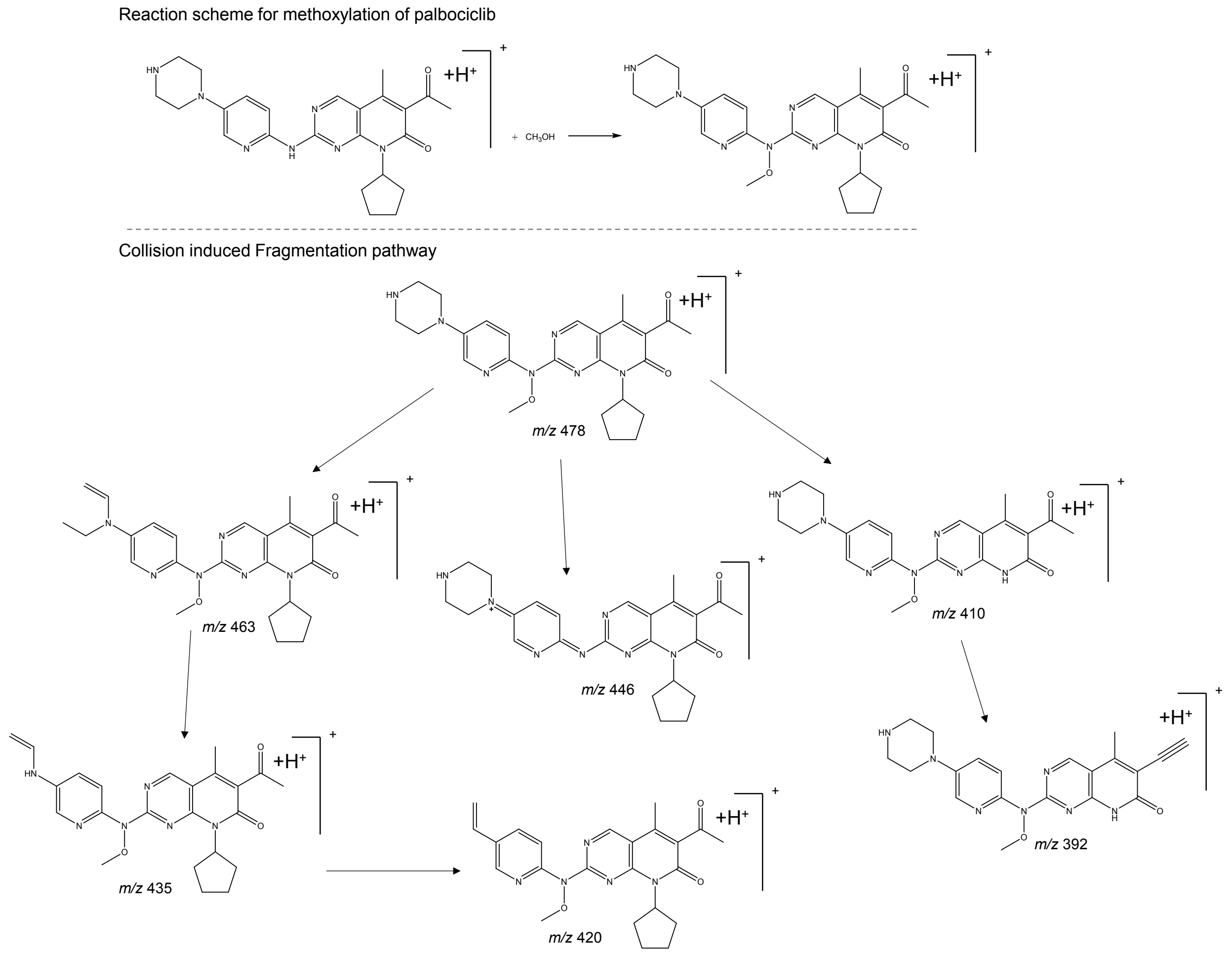

Figure S18: Formation and fragmentation pathway of methoxylated palbociclib detected at *m/z* 478.

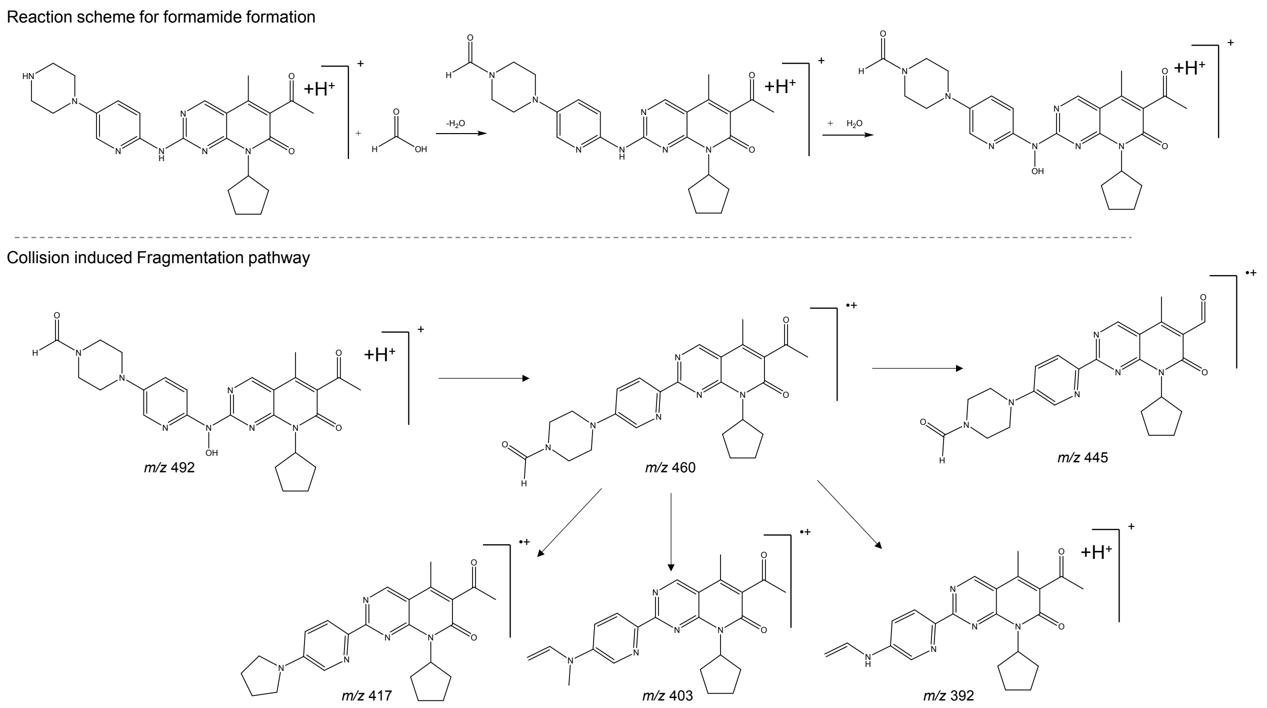

Figure S19: Formation and fragmentation pathway of the product upon reaction with formic acid and palbociclib leading to a peak at *m/z* 492.

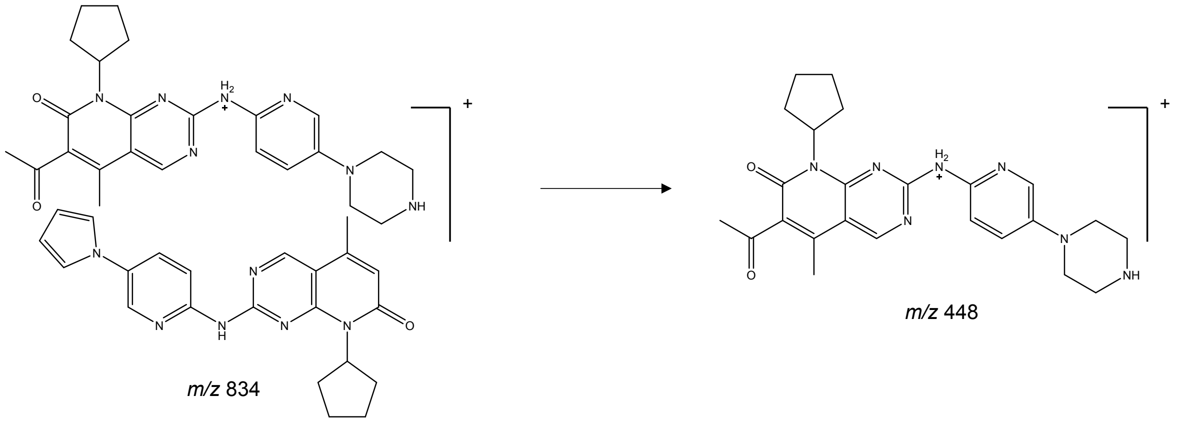

Figure S20: Formation of the ion adduct detected at *m/z* 834.

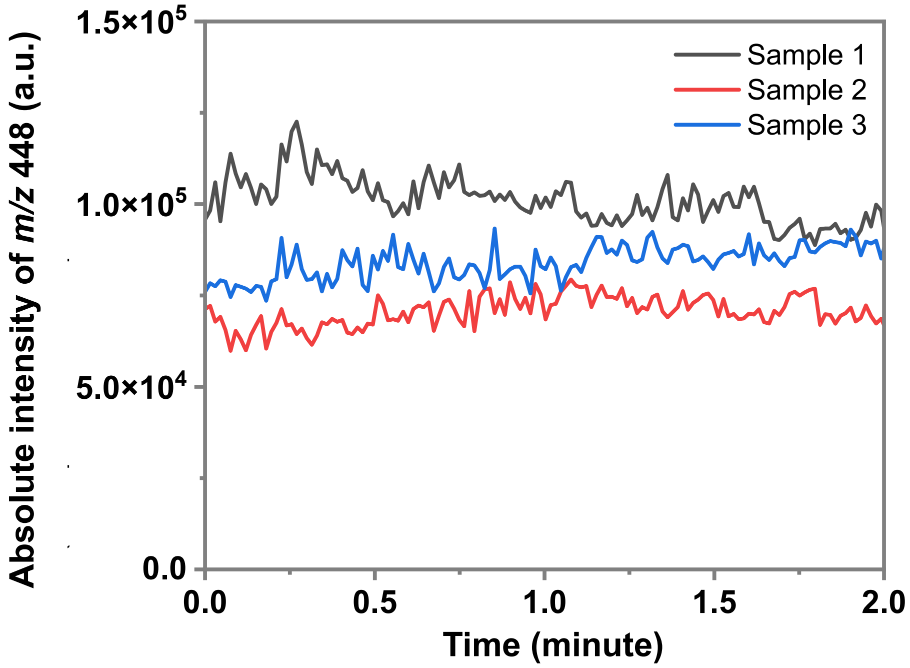

Figure S21. Comparative extracted ion chromatograms from chicken tissue samples spiked with 100 µM palbociclib. The CFPSI instrument equilibrium time has been subtracted from the total acquisition time. The three chronograms indicate the stable signal intensity of *m/z* 448 over time across different portions of the same tissue.

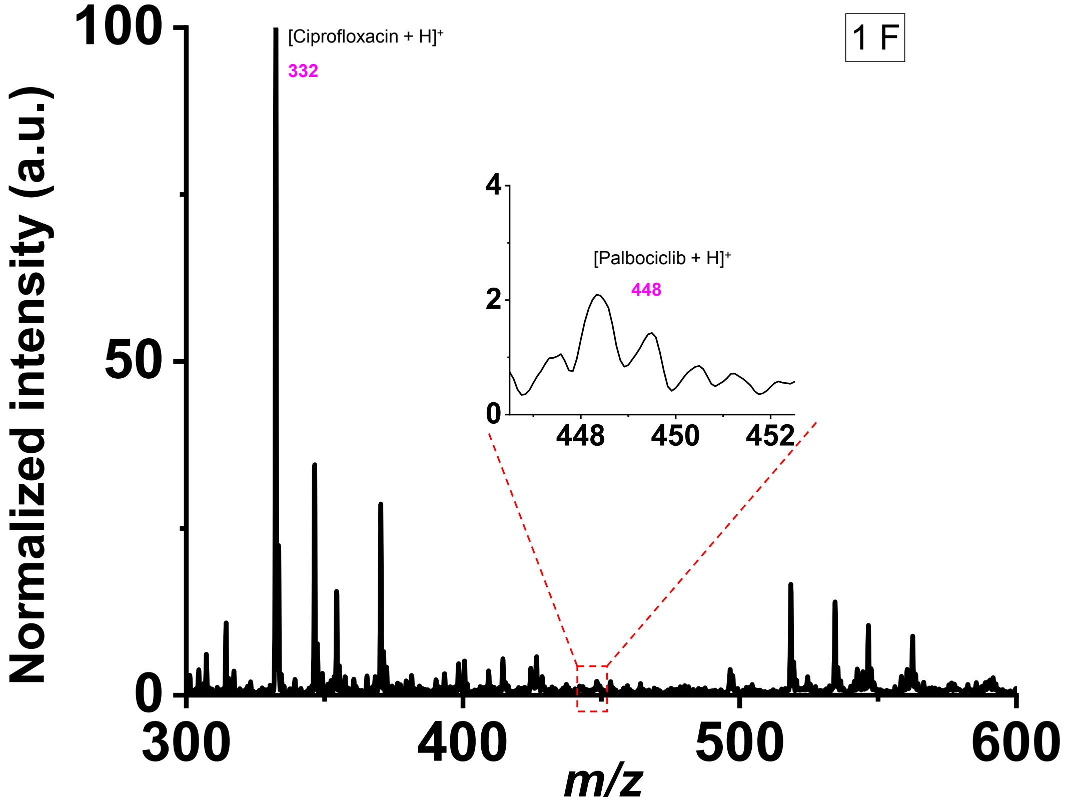

Figure S22. CFPSI MS spectrum of PDX model tissue samples displaying the presence of a lower concentration of palbociclib. The concentration found from the calibration curve is ~32 nM.

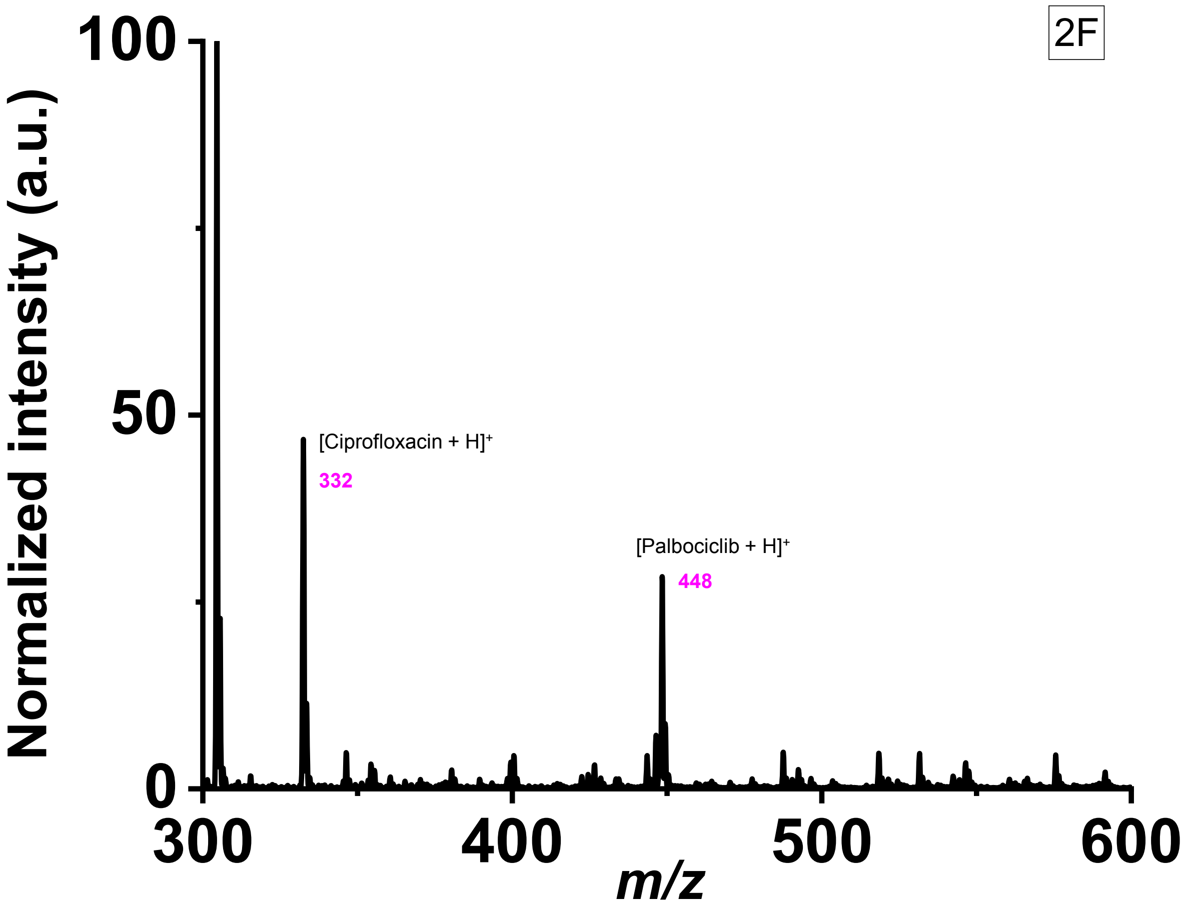

Figure S23. CFPSI MS spectrum of PDX model tissue samples displaying the presence of a lower concentration of palbociclib. The concentration found from the calibration curve is ~31 µM.

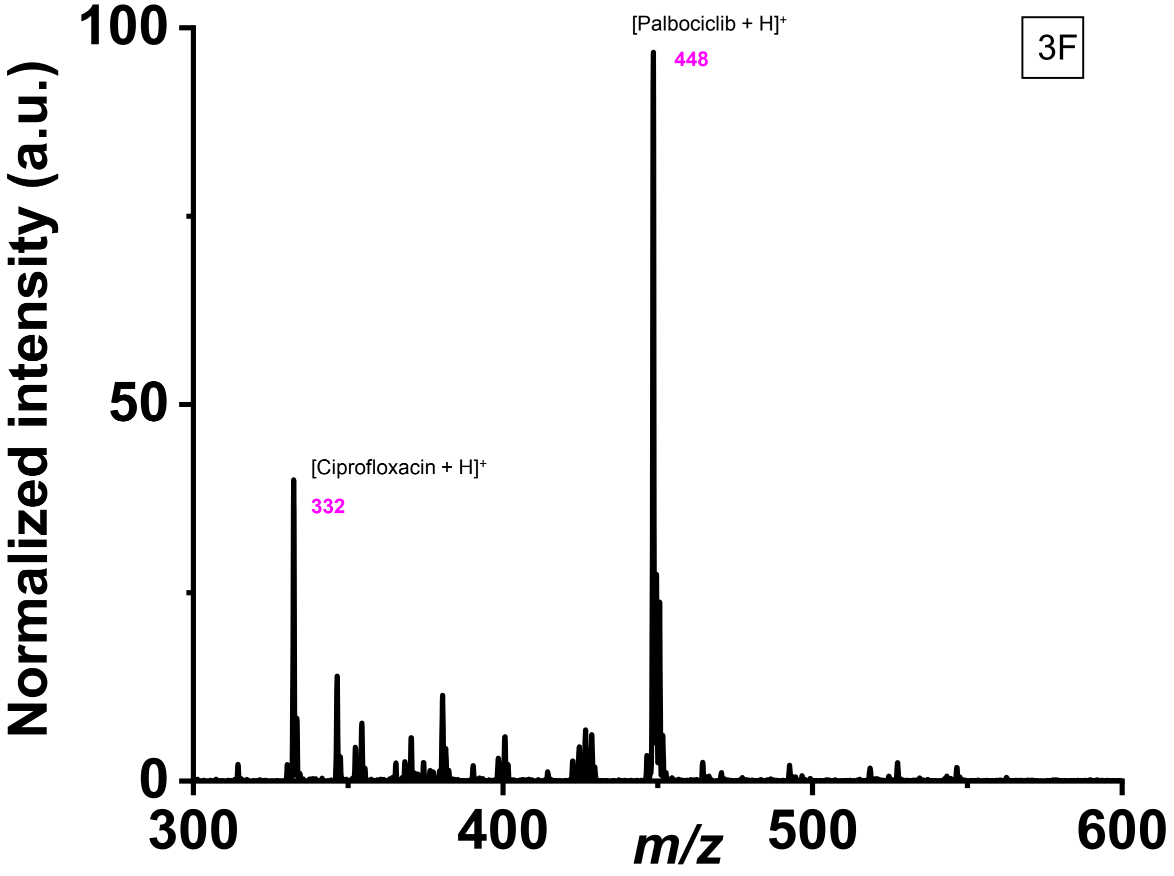

Figure S24. CFPSI MS spectrum of PDX model tissue samples displaying the presence of a lower concentration of palbociclib. The concentration found from the calibration curve is ~132 µM.

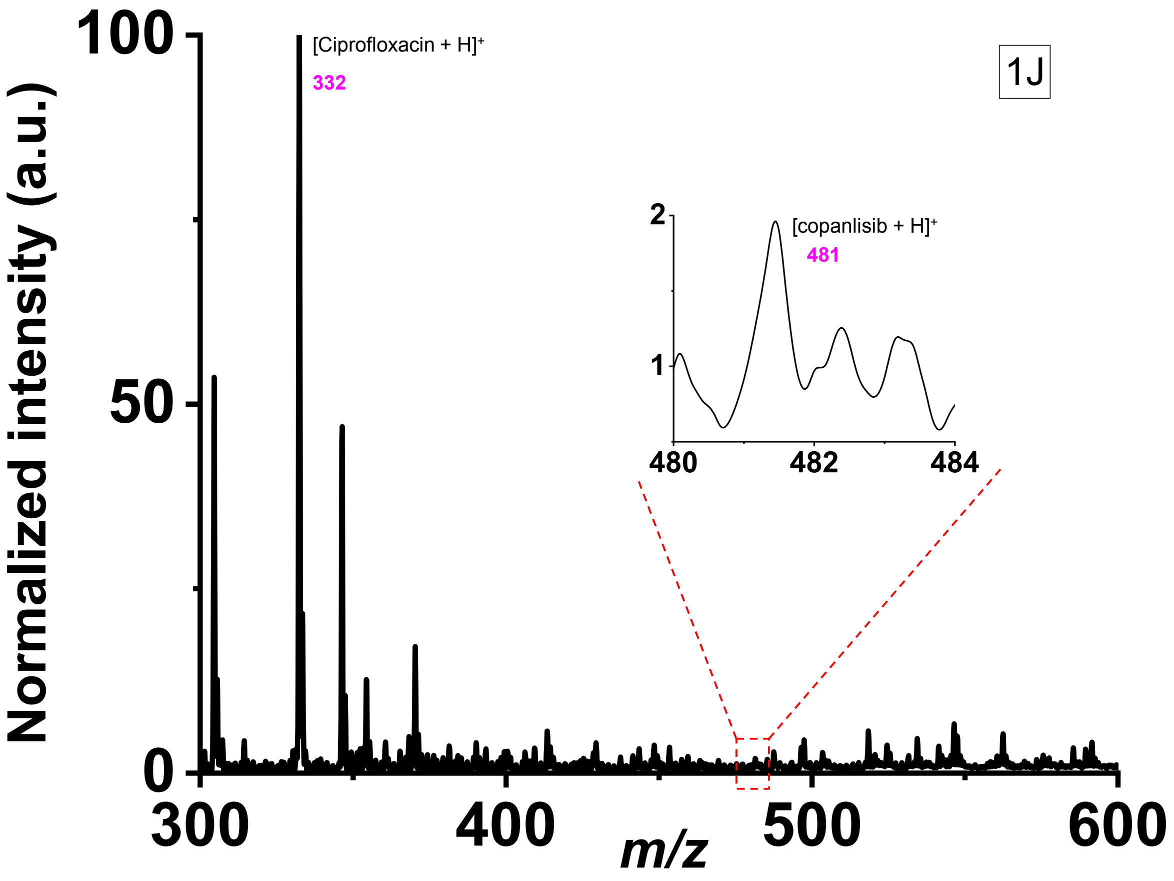

Figure S25. CFPSI MS spectrum of PDX model tissue samples displaying the presence of a lower concentration of palbociclib. The concentration found from the calibration curve is ~11 nM.

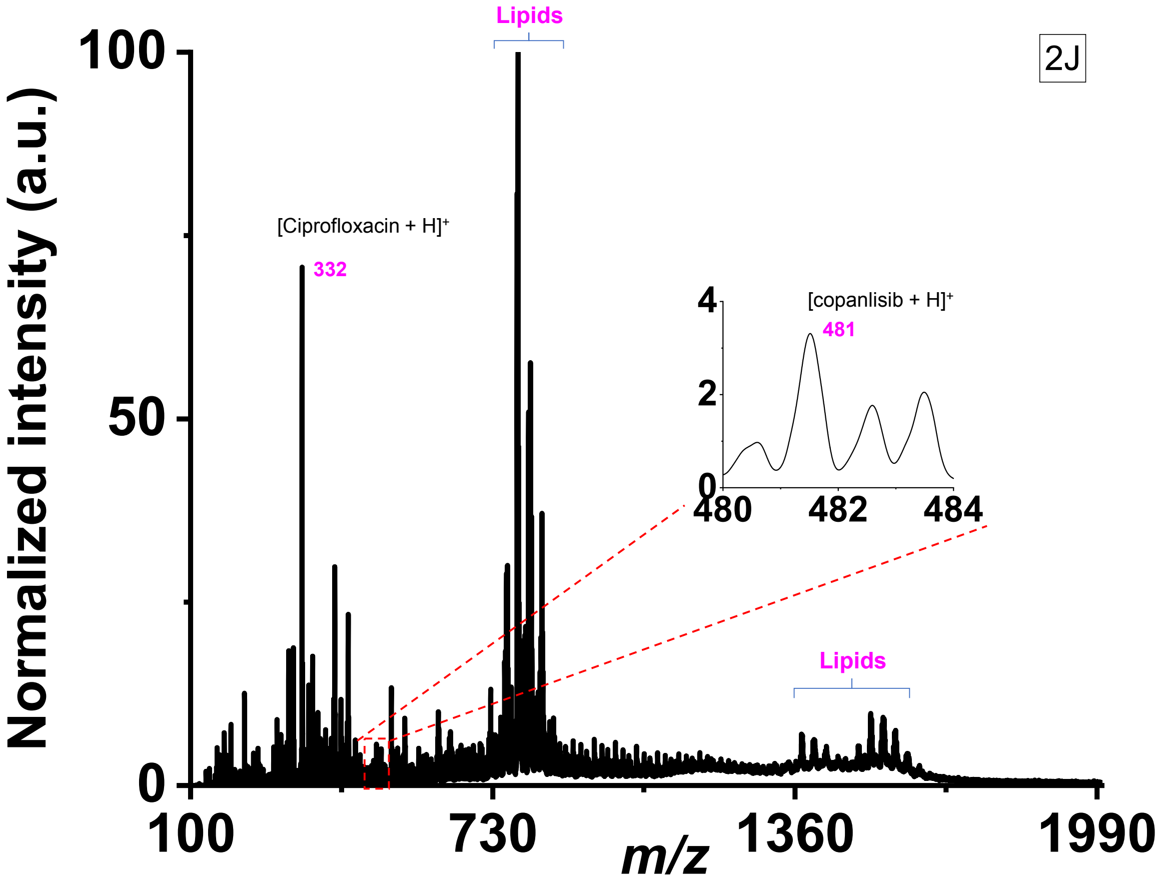

Figure S26. CFPSI MS spectrum of PDX model tissue samples displaying the presence of a lower concentration of palbociclib. The concentration found from the calibration curve is ~65 nM.

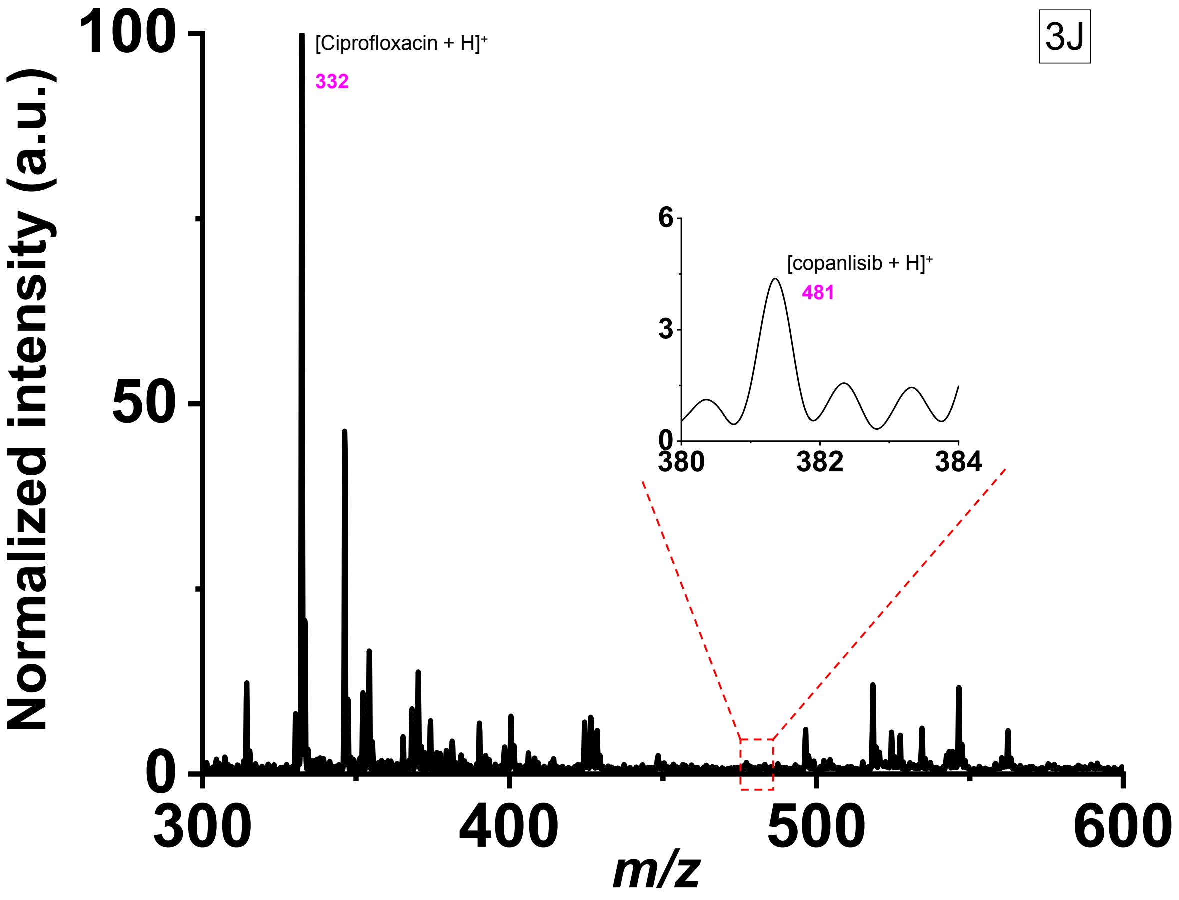

Figure S27. CFPSI MS spectrum of PDX model tissue samples displaying the presence of a lower concentration of palbociclib. The concentration found from the calibration curve is ~160 nM.
